# A new model for regulation of sphingosine kinase 1 translocation to the plasma membrane in breast cancer cells

**DOI:** 10.1016/j.jbc.2021.100674

**Published:** 2021-04-16

**Authors:** Ryan D.R. Brown, Ben E.P. Veerman, Jeongah Oh, Rothwelle J. Tate, Federico Torta, Margaret R. Cunningham, David R. Adams, Susan Pyne, Nigel J. Pyne

**Affiliations:** 1 Strathclyde Institute of Pharmacy and Biomedical Sciences, University of Strathclyde, Glasgow, Scotland, UK; 2 SLING, Singapore Lipidomics Incubator, Life Sciences Institute and Department of Biochemistry, YLL School of Medicine, National University of Singapore, Singapore, Singapore; 3 School of Engineering & Physical Sciences, Heriot-Watt University, Edinburgh, UK

**Keywords:** Sphingosine-1-phosphate (S1P), translocation, plasma membrane, mutagenesis *in vitro*, fluorescence, structural biology, lipid signaling, enzyme mechanism, AUC, area under the curve, BuMe, butanol:methanol, CTD, C-terminal domain, DAPI, 4′,6-diamidino-2-phenylindole, ERK, extracellular signal–regulated protein kinase, FCS, fetal calf serum, FIPI, *N*-[2-[4-(2,3-dihydro-2-oxo-1*H*-benzimidazol-1-yl)-1-piperidinyl]ethyl]-5-fluoro-1*H*-indole-2-carboxamide hydrochloride, *h*SK1, human SK1, LBL-1, lipid-binding loop 1, *m*SK1, mouse SK1, NTD, N-terminal domain, PA, phosphatidic acid, PLAs, proximity ligation assays, PLD, phospholipase D, PLD2, phospholipase D2, PM, plasma membrane, PMA, phorbol 12-myristate 13-acetate, PS, phosphatidylserine, R-loop, regulatory loop, S1P, sphingosine-1-phosphate, SK1, sphingosine kinase 1, Sph, sphingosine

## Abstract

The translocation of sphingosine kinase 1 (SK1) to the plasma membrane (PM) is crucial in promoting oncogenesis. We have previously proposed that SK1 exists as both a monomer and dimer in equilibrium, although it is unclear whether these species translocate to the PM *via* the same or different mechanisms. We therefore investigated the structural determinants involved to better understand how translocation might potentially be targeted for therapeutic intervention. We report here that monomeric WT mouse SK1 (GFP-*m*SK1) translocates to the PM of MCF-7L cells stimulated with carbachol or phorbol 12-myristate 13-acetate, whereas the dimer translocates to the PM in response to sphingosine-1-phosphate; thus, the equilibrium between the monomer and dimer is sensitive to cellular stimulus. In addition, carbachol and phorbol 12-myristate 13-acetate induced translocation of monomeric GFP-*m*SK1 to lamellipodia, whereas sphingosine-1-phosphate induced translocation of dimeric GFP-*m*SK1 to filopodia, suggesting that SK1 regulates different cell biological processes dependent on dimerization. GFP-*m*SK1 mutants designed to modulate dimerization confirmed this difference in localization. Regulation by the C-terminal tail of SK1 was investigated using GFP-*m*SK1 truncations. Removal of the last five amino acids (PPEEP) prevented translocation of the enzyme to the PM, whereas removal of the last ten amino acids restored translocation. This suggests that the penultimate five amino acids (SRRGP) function as a translocation brake, which can be released by sequestration of the PPEEP sequence. We propose that these determinants alter the arrangement of N-terminal and C-terminal domains in SK1, leading to unique surfaces that promote differential translocation to the PM.

Sphingosine-1-phosphate (S1P) is a bioactive lipid that is formed by the phosphorylation of sphingosine (Sph) by sphingosine kinase, of which there are two isoforms (sphingosine kinase 1 [SK1] and sphingosine kinase 2). SK1 and sphingosine kinase 2 are encoded by different genes and have distinct subcellular localization and biochemical properties (1). S1P is degraded by S1P lyase, to produce (*E*)-2-hexadecenal and phosphoethanolamine, and by S1P phosphatase, which dephosphorylates S1P to form Sph (1). S1P is released from cells *via* specific transporters in the plasma membrane (PM) and then binds to and stimulates a family of G protein–coupled receptors, the S1P receptors (S1P_1_-S1P_5_), on cells or can act on intracellular targets, such as histone deacetylase 1/2, to induce cellular responses (1). An important role for S1P in cancer is evident from studies showing that high expression of SK1 and S1P receptors in tumors is linked with poor prognosis in patients (2, 3, 4). S1P also promotes transformation, epithelial mesenchymal transition and invasiveness, cancer cell survival, replicative immortality, tumor neovascularization, and aerobic glycolysis—the so-called hallmarks of cancer (1, 5, 6). Therefore, SK1 is a target for therapeutic intervention in cancer. Indeed, oncogenic transformation of NIH3T3 cells is induced by overexpression of SK1 (7), and this involves its translocation from the cytoplasm to the PM, a process that allows access to the substrate (Sph). The importance of this process is evident from studies showing that the overexpression of a kinase-dead G82D *h*SK1 mutant fails to induce transformation, thereby demonstrating dependency on the catalytic activity of SK1 and therefore S1P (7).

A key step in the transformation process is the phosphorylation of SK1 by extracellular signal–regulated kinase (ERK) on Ser225 in *h*SK1, which promotes translocation of the enzyme from the cytoplasm to the PM (8, 9). The importance of this is underlined by the fact that the constitutive localization of SK1 to the PM promotes oncogenic transformation (9). Moreover, studies have demonstrated that SK1 activation and localization to the PM and subsequent stimulation of the S1P_2_ receptor by released S1P (‘inside-out’ signaling) increase transferrin receptor 1 expression (10), and this is critically important in promoting transformation, as evidenced by data showing that a neutralizing anti–transferrin receptor 1 antibody blocks oncogenesis (10).

Dynamically regulated localization of SK1 to the PM is clearly important for the cellular function of the enzyme. Although the structural basis for this controlled targeting remains incompletely understood at present, the emergence of crystal structures for SK1 has begun to shed some light on the issue in recent years. The enzyme adopts a bidomain organization with a C-terminal domain (CTD) that hosts the lipid substrate binding site and an N-terminal domain (NTD) that binds ATP; the catalytic center is formed at the interface between these two domains (as reviewed (11)). Sph is bound within a J-shaped cavity (the ‘J-channel’), formed by packing of three extended lipid-binding loop structures against a β-sandwich core in the CTD. An exposed hydrophobic patch on the exterior of lipid-binding loop 1 (LBL-1) (L194/F197/L198 in human SK1 [*h*SK1]) has been shown to be a key determinant for membrane engagement, including curvature-sensitive binding (12, 13). The same hydrophobic patch on LBL-1 has also been implicated in the binding of SK1 to calcium- and integrin-binding protein, a calcium-sensing partner protein that is involved in the translocation of SK1 from the cytoplasm to the membrane (14).

Acidic phospholipids also play a critical role in the membrane localization of SK1, notably phosphatidylserine (PS) and phosphatidic acid (PA) (15, 16, 17). Early studies implicated Thr54 and Asn89 of *h*SK1 in the interaction of the enzyme with PS (15, 18), but recent attention has focused on a set of basic residues as a candidate interface for acidic phospholipid engagement. The interface was first identified by analysis of *h*SK1 crystal structures (11). In all five of the currently available structures, the protein adopts a dimeric assembly (19, 20, 21), consistent with coimmunoprecipitation analysis that showed that SK1 can dimerize (22). The mode of dimerization generates a pronounced groove at the dimer interface that is aligned parallel with the LBL-1 loops of the two protomers within the assembly. The grooved surface exhibits a pronounced positive electrostatic potential surface because of contributions from basic residues within the NTD and on the tip of LBL-1, and this is topographically coordinated with the LBL-1 hydrophobic patches to provide a suitable membrane-binding interface (11). Recent mutagenesis studies have validated the proposed membrane engagement surface and specifically identified K27, K29, and R186 in *h*SK1 as residues that are important for binding to PA-enriched target membranes (13).

The Ser225 phosphorylation site for ERK in *h*SK1 (conserved in mouse SK1 [*m*SK1]) occupies a prominently solvent-exposed position within an extended regulatory loop (the ‘R-loop’) that packs on the reverse side of the CTD β-sandwich core to LBL-1 and is therefore on the opposite side of the protein to the membrane engagement surface. The mechanism underpinning translocation in response to phosphorylation at this site has not yet been elucidated. However, it may involve induced interdomain movement that affects the presentation of key basic residues in the NTD relative to LBL-1 in the CTD and thence whether the protein presents a topographically coordinated combination of surface elements or misaligned patches; R-loop phosphorylation might also impact on the capacity of SK1 to adopt a dimeric assembly in principle (11). As an added complexity, there are additional drivers for translocation of SK1 to the PM that are independent of S225 phosphorylation in SK1. For example, G_q_ has been shown to promote translocation of *m*SK1 and *h*SK1 to the PM in HEK293 cells in a manner that is dependent on the generation of the activated Gα_q_ subunit but independent of downstream [Ca^2+^]_i_ elevation or phosphorylation by ERK (23).

The C-terminal tail of SK1 binds proteins, including tumor necrosis factor receptor-associated factor 2 (24) and protein phosphatase 2 (25). We have suggested that protein binding to the C-terminal tail might have an important role in regulating translocation of SK1 to the PM (11). Indeed, truncation immediately preceding Gly364 in *h*SK1 to remove the C-terminal tail (21 residues) has been shown to promote constitutive activation and binding to PS-containing lipid raft microdomains (26) in a manner independent of ERK phosphorylation.

The existence of both phosphorylation-dependent and phosphorylation-independent mechanisms for translocation of SK1 from the cytoplasm to the PM raises a question as to whether there is any mechanistic intersection between the differing modes of translocation at the level of protein structure. This might occur, for example, if the ERK phosphorylation destabilizes an autoinhibitory C-terminal folding so as to correctly present regions required for binding acidic phospholipids. This would represent a unified mechanism for membrane localization, promoted either by ERK phosphorylation or by alteration of the position of the C-terminal tail. We have therefore investigated the structural/functional properties of SK1 that facilitate its translocation from the cytoplasm to the PM of MCF-7L breast cancer cells using *m*SK1. This includes defining whether the monomeric or dimeric forms of SK1 localize to different microdomains in the PM, potentially to facilitate distinct aspects of the enzyme’s pleiotropic signaling functions, and defining the role of the C-terminal tail of SK1 in regulating translocation. Such studies inform on evolutionary conserved structural–functional properties of SK1 and enable identification of possible deregulated mechanisms controlling translocation of SK1 in preclinical murine disease models.

## Results

### Translocation of SK1 to different microdomains in the PM

**Figure 1:**
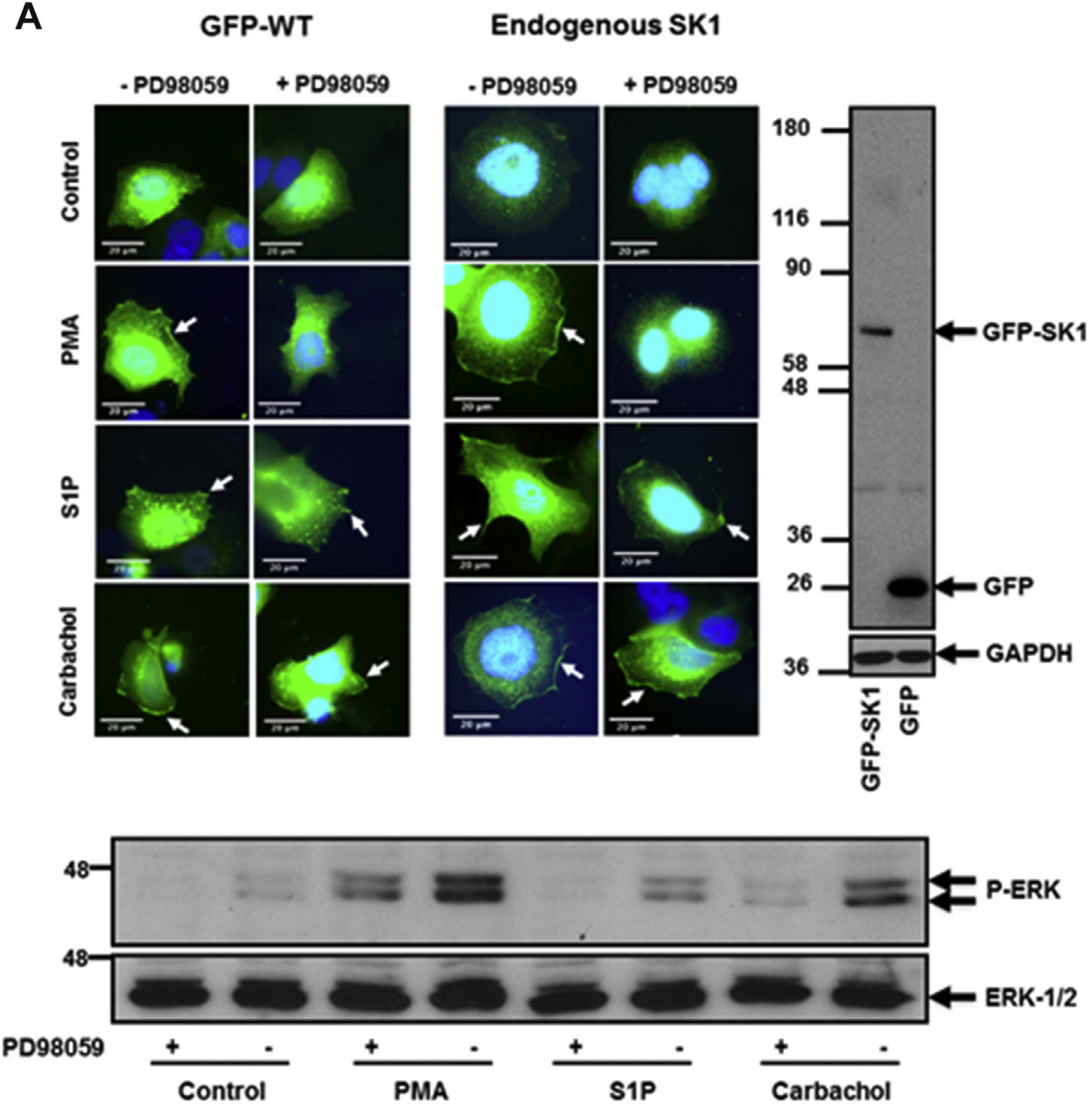

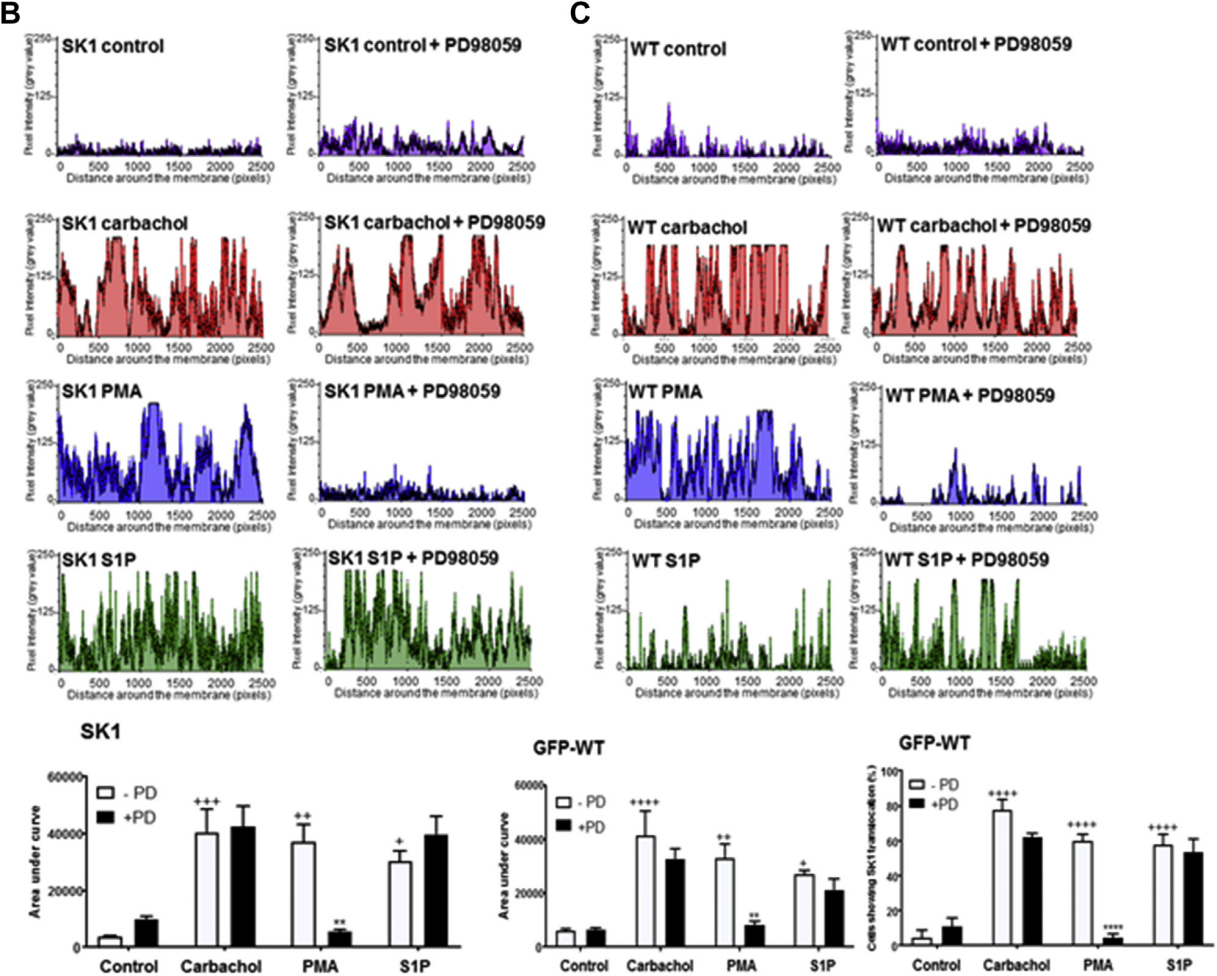

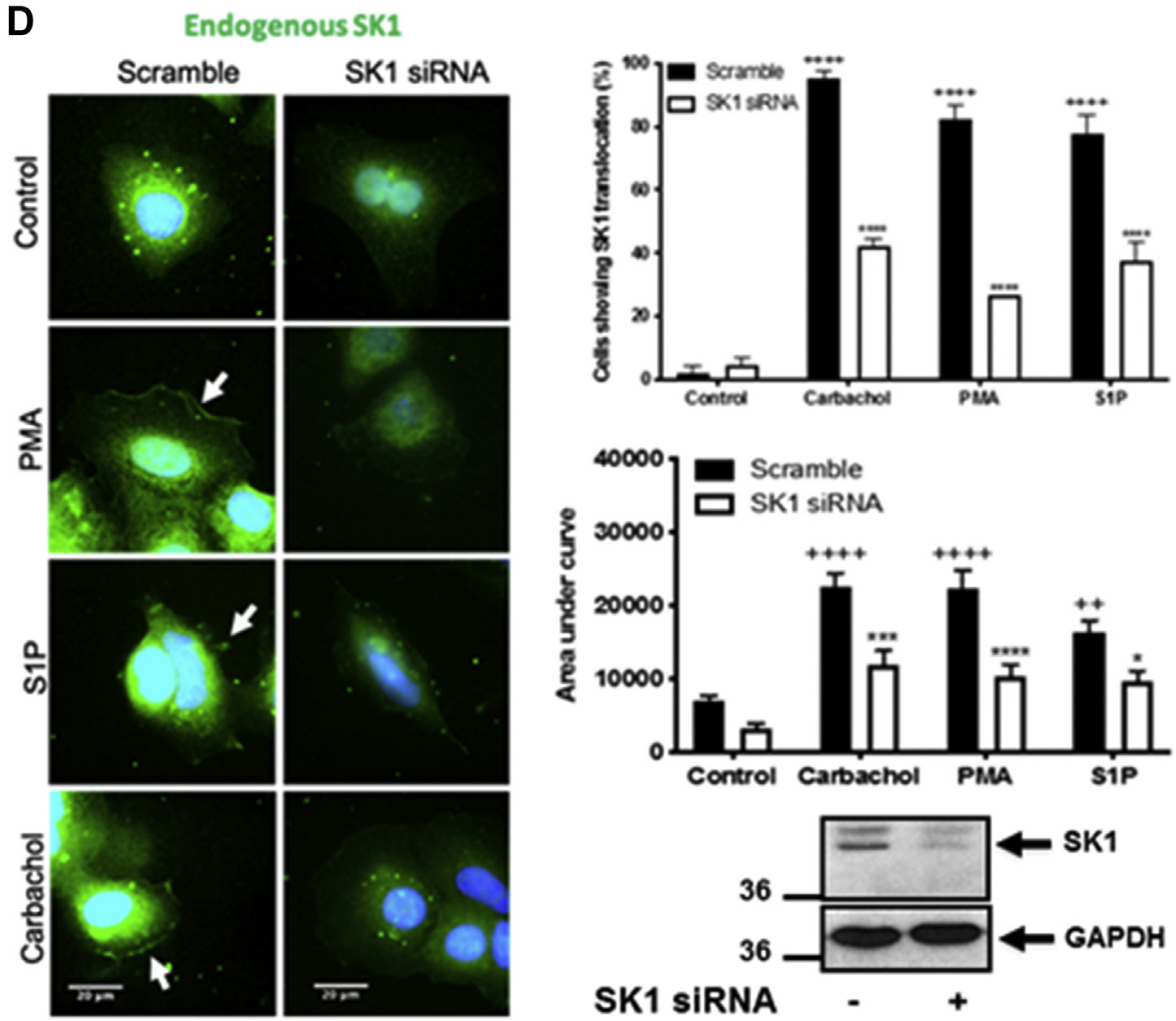
**Effect of the MEK-1 inhibitor, PD98059, on the translocation of endogenous SK1 and WT GFP-*m*SK1 in MCF-7L cells.** MCF-7L cells expressing endogenous SK1 and WT GFP-*m*SK1 were pretreated with and without PD98059 (50 μM, 1 h) or SK1 siRNA (200 nM, 24 h) before S1P (5 μM) or PMA (1 μM,) or carbachol (100 μM) for 10 min. Cells were processed (see Experimental procedures) and mounted with DAPI to stain the DNA (*blue*). *A*, photomicrographs of cells of 40× oil magnification expressing endogenous SK1 or WT GFP-*m*SK1 (detected with an anti-SK1 antibody and FITC-conjugated secondary antibody or by GFP immunofluorescence, respectively). Inset is a Western blot probed with the anti-GFP antibody or P-ERK antibodies showing the overexpression of WT GFP-*m*SK1 and inhibition of ERK-1/2 phosphorylation by PD98059. Reprobing with total ERK-1/2 or GAPDH is used to confirm similar protein loading. Representative results of three independent experiments. *B* and *C*, membrane intensity measurements were made from five individual MCF-7L cells and stitched together (see Experimental procedures) (n = 5) for endogenous SK1 (*B*) and WT GFP-*m*SK1 (*C*). *B*, the bar graph represents the AUC of the total level of endogenous SK1 translocation (n = 5); ^∗^^∗^*p* < 0.01 PMA alone *versus* PMA with PD98059; ^+^*p* < 0.05, ^++^*p* < 0.01, and ^+++^*p* < 0.001 for stimulus *versus* control for endogenous SK1 (two-way ANOVA with Tukey's post hoc test). *C*, the *left bar graph* represents the AUC of transfected WT GFP-*m*SK1 translocation (n = 5). The *right bar graph* represents the percentage of cells containing translocated WT GFP-*m*SK1 (n = 3); ^∗^^∗^*p* < 0.01, ^∗^^∗^^∗^^∗^*p* < 0.0001 PMA alone *versus* PMA with PD98059; ^+^*p* < 0.05, ^++^*p* < 0.01, and ^++++^*p* < 0.0001 for stimulus *versus* control transfected WT GFP-*m*SK1 (two-way ANOVA with Tukey's post hoc test). *D*, photomicrographs of cells of 40× oil magnification showing the effect of SK1 siRNA on endogenous SK1 expression. The *upperbar graph* represents the percentage of cells (n = 3) containing translocated endogenous SK1. ^++++^*p* < 0.0001 for control scrambled *versus* stimulated scrambled and ^∗^^∗^^∗^*p* < 0.001 for SK1 siRNA *versus* scrambled (two-way ANOVA with Tukey's post hoc test). The *lowerbar graph* represents the AUC of the total level of endogenous SK1 translocation (n = 5). ^++^*p* < 0.01 and ^++++^*p* < 0.0001 for control scrambled *versus* stimulated scrambled, ^∗^*p* < 0.05, ^∗^^∗^^∗^*p* < 0.001, and ^∗^^∗^^∗^^∗^*p* < 0.0001 for SK1 siRNA *versus* scrambled (two-way ANOVA with Tukey's post hoc test). AUC, area under the curve; ERK, extracellular signal–regulated kinase; *m*SK1, mouse SK1; PMA, phorbol 12-myristate 13-acetate; SK1, sphingosine kinase 1.

Confirmation of the expression of WT GFP-*m*SK1 mutants was by Western blot analysis using the anti-GFP antibody (Fig. 1*A*). The treatment of MCF-7L cells with S1P (5 μM, 10 min), phorbol 12-myristate 13-acetate (PMA) (1 μM, 10 min), or carbachol (100 μM, 10 min) induced translocation of endogenous (detected with the anti-SK1 antibody) or WT GFP-*m*SK1 from the cytoplasm to the PM (Fig. 1*A*). Carbachol binds to M_1_- and G_q_-coupled M_3_ muscarinic receptors in MCF-7 cells (27); S1P has been shown to bind to G_q_-coupled S1P_3_ in MCF-7 cells (28), and S1P, PMA, or carbachol activate phospholipase D (PLD) to form PA (29, 30, 31). Translocation of SK1 was quantified by measuring membrane immunoreactivity or GFP intensity at the PM as an area under the curve (AUC) as described in Experimental procedures. Both endogenous SK1 and WT GFP-*m*SK1 exhibited significant increases in the AUC at the PM after treatment with ligands (Fig. 1, *B* and *C*). Results were also quantified as the percentage of cells containing PM-associated SK1 as described in Experimental procedures. In WT GFP-*m*SK1 transiently overexpressing MCF-7L cells, there appeared to be very little SK1 associated with the PM, with the majority of SK1 localized in the cytoplasm and perinuclear region. Treatment of cells with S1P, PMA, or carbachol stimulated translocation of WT GFP-*m*SK1, such that there was an increase in the percentage of cells containing PM SK1 (Fig. 1, *B* and *C*). These responses were observed as a redistribution of SK1 from the cytoplasm/perinuclear regions to the PM. GFP-transfected cells stimulated with PMA, carbachol, or S1P did not show a redistribution of GFP (Fig. S1). To confirm the identity of the endogenous *h*SK1, MCF-7L cells were treated with SK1 siRNA, which reduced the intensity of the immunostaining of a 42-kDa protein detected on Western blot of cell lysates and decreased the immunofluorescence at the PM in response to ligand stimulation of the cells (Fig. 1*D*).

### Dependency on ERK phosphorylation

We next investigated whether the translocation of SK1 to the PM might be *via* an ERK-catalyzed phosphorylation-dependent or phosphorylation-independent mechanism in MCF-7L breast cancer cells. In this case, the pretreatment of MCF-7L cells with the MEK-1 inhibitor, PD98059 (50 μM, 1 h), decreased activation of ERK-1/2 to all three ligands (Fig. 1*A*) and reduced the translocation of both endogenous SK1 and WT GFP-*m*SK1 in response to PMA, but not carbachol or S1P (Fig. 1, *A*–*C*). This difference in sensitivity to ERK might be due to the finding that PMA is a stronger activator of ERK than carbachol or S1P. These findings are supported by studies showing that *m*SK1 can be phosphorylated by ERK (8). Therefore, carbachol and S1P are ligands that appear to induce the translocation of SK1 to the PM *via* an ERK-independent mechanism in MCF-7L cells.

### Colocalization studies

**Figure 2:**
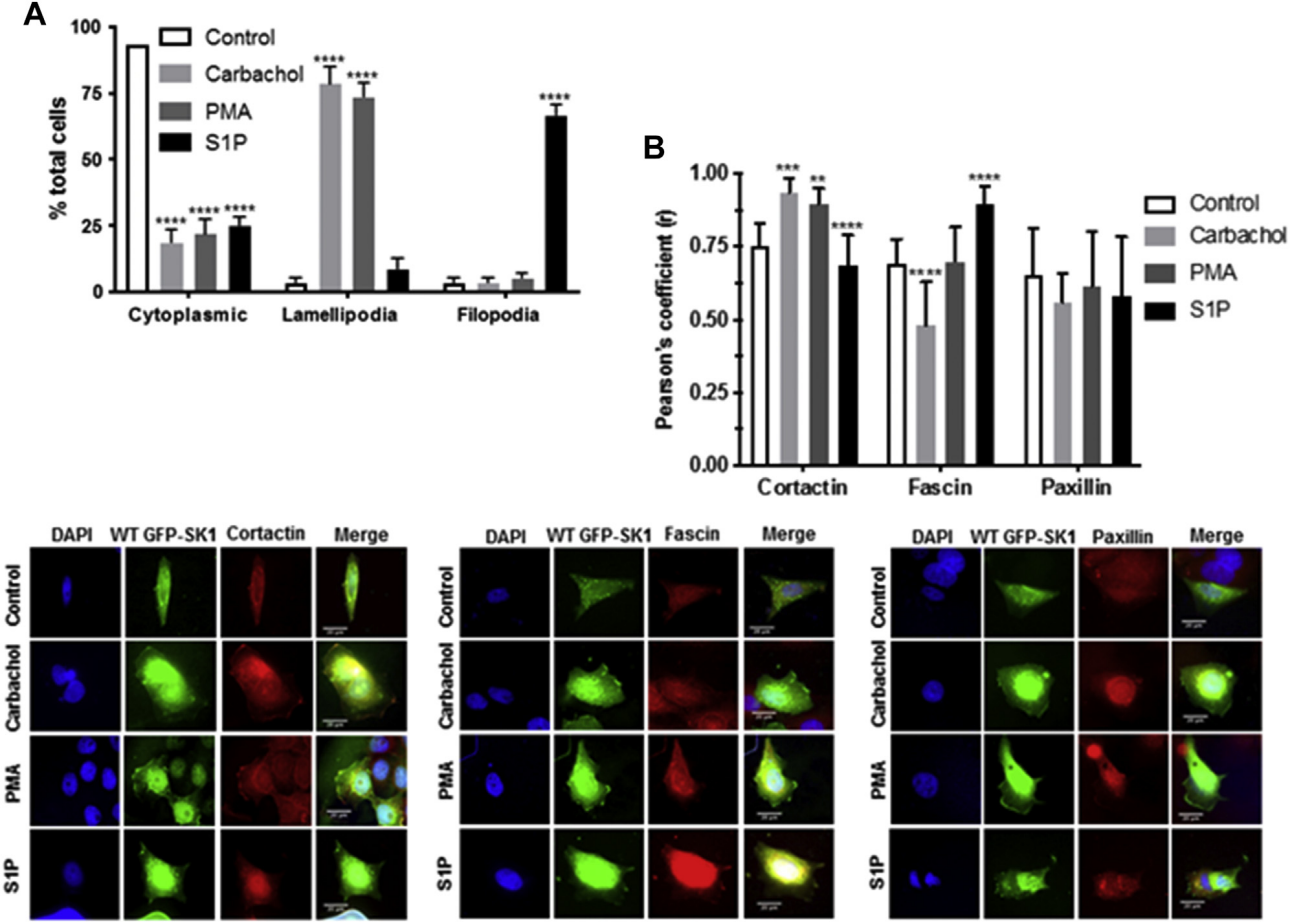
**Colocalization of SK1 with markers of lamellipodia and filopodia.** MCF-7L cells overexpressing WT GFP-mSK1 were treated with S1P (5 μM) or PMA (1 μM) or carbachol (100 μM) for 10 min. Cells were processed and mounted with DAPI to stain the DNA (*blue*). *A*, the bar graph representing the percentage of cells containing translocated WT GFP-*m*SK1 in lamellipodia and filopodia microdomains in the PM in response to stimulus (n = 4); ^∗^^∗^^∗^^∗^*p* < 0.0001 treated *versus* control (two-way ANOVA with Tukey's post hoc test). *B*, photomicrographs of cells of 40× oil magnification overexpressing WT GFP-*m*SK1 detected with GFP and cortactin (marker of lamellipodia), fascin (marker of filopodia), and paxillin (marker of adhesion foci) detected with respective specific antibodies. Representative results of three independent experiments. Also shown is a bar graph of the Pearson Correlation Coefficients of colocalization. ^∗^^∗^*p* < 0.01, ^∗^^∗^^∗^*p* < 0.001, and ^∗^^∗^^∗^^∗^*p* < 0.0001 for Pearson Correlation Coefficients for stimulated *versus* control (increase/decrease) using two-way ANOVA with Tukey's post hoc test. *m*SK1, mouse SK1; PM, plasma membrane; PMA, phorbol 12-myristate 13-acetate; S1P, sphingosine-1-phosphate; SK1, sphingosine kinase 1.

The treatment of cells with PMA or carbachol resulted in the translocated SK1 being evenly distributed (spread) in the PM in substructures reminiscent of lamellipodia (Fig. 2*A*). In contrast, S1P promoted translocation of SK1 to focal regions in the PM (Fig. 2*A*). To better define the PM substructures in which WT GFP-*m*SK1 is translocated, colocalization studies were performed using defined markers of lamellipodia (cortactin), filopodia (fascin), and focal adhesions (paxillin). Pearson correlation coefficients indicated that carbachol and PMA stimulated the colocalization of WT GFP-*m*SK1 with cortactin (Fig. 2*B*), indicating localization of lamellipodia, while S1P promoted the colocalization of WT GFP-*m*SK1 with fascin (Fig. 2*B*), indicating localization of filopodia. There was no colocalization of GFP-*m*SK1 with paxillin (Fig. 2*B*), indicating that the substructure localization does not include focal adhesions.

### Dependency on PLD1/2 and G_q_

**Figure 3:**
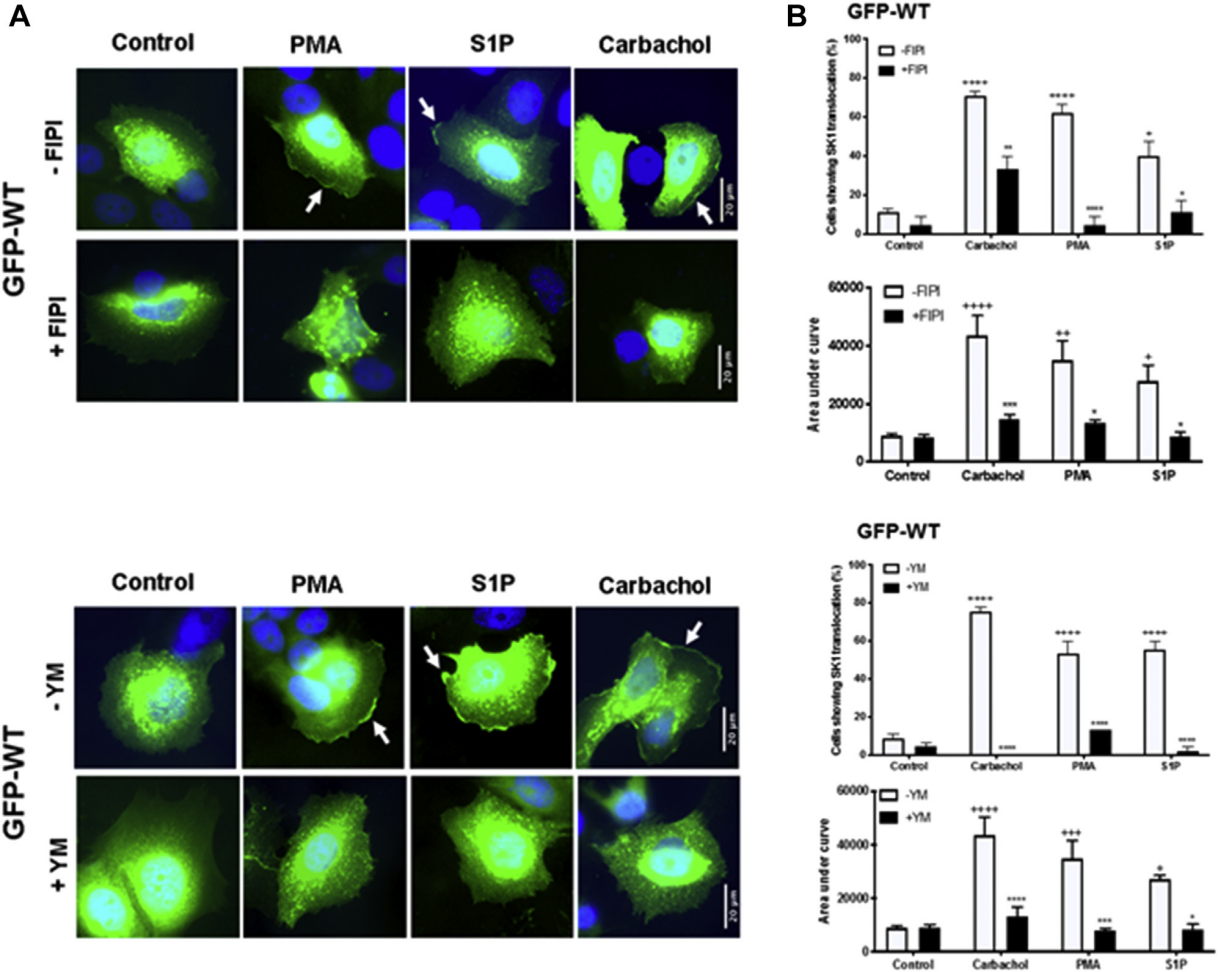
**Effect of the PLD inhibitor, FIPI, or the G**_**q**_**inhibitor YM254890 on the translocation of WT GFP-*m*SK1 in MCF-7L cells.** MCF-7L cells overexpressing WT GFP-*m*SK1 were pretreated with and without FIPI (100 nM, 1 h) or YM254890 (10 μM, 30 min) before S1P (5 μM) or PMA (1 μM) or carbachol (100 μM) for 10 min. Cells were processed and mounted with DAPI to stain the DNA (*blue*). *A*, photomicrographs of cells of 40× oil magnification overexpressing WT GFP-*m*SK1 detected with GFP. Representative results of three independent experiments. *B*, the bar graphs (*lower of each pair*) represent the AUC of transfected WT GFP-*m*SK1 translocation (n = 5) and (*upper of each pair*) the percentage of cells containing translocated WT GFP-*m*SK1 (n = 3); ^∗^*p* < 0.05, ^∗^^∗^*p* < 0.01, ^∗^^∗^^∗^*p* < 0.001, and ^∗^^∗^^∗^^∗^*p* < 0.0001 for stimulus alone *versus* stimulus with either FIPI or YM254890; ^+^*p* < 0.05, ^++^*p* < 0.01, ^+++^*p* < 0.001, and ^++++^*p* < 0.0001 for stimulus *versus* control transfected WT GFP-*m*SK1 (two-way ANOVA with Tukey's post hoc test). AUC, area under the curve; FIPI, *N*-[2-[4-(2,3-dihydro-2-oxo-1*H*-benzimidazol-1-yl)-1-piperidinyl]ethyl]-5-fluoro-1*H*-indole-2-carboxamide hydrochloride; *m*SK1, mouse SK1; PLD, phospholipase D; PMA, phorbol 12-myristate 13-acetate; S1P, sphingosine-1-phosphate; SK1, sphingosine kinase 1.

To establish whether the translocation of WT GFP-*m*SK1 in MCF-7L cells is dependent on formation of acidic phospholipids, MCF-7L cells were pretreated with the PLD1/2 inhibitor, *N*-[2-[4-(2,3-dihydro-2-oxo-1*H*-benzimidazol-1-yl)-1-piperidinyl]ethyl]-5-fluoro-1*H*-indole-2-carboxamide hydrochloride [FIPI]. PLD catalyzes the hydrolysis of phosphatidylcholine to produce acidic PA and choline. Treatment of MCF-7L cells with FIPI (100 nM, 1 h) reduced the translocation of WT GFP-*m*SK1 in response to carbachol, PMA, or S1P (Fig. 3, *A* and *B*). This likely reflects a role for PKC activation, directly (in the case of PMA) or indirectly (as a consequence of G_q_/PLC signaling), in the stimulation of PLD and generation of PA. To confirm the involvement of G_q_, MCF-7L cells were pretreated with the G_q_ inhibitor, YM254890, which inhibits GDP–GTP exchange. YM254890 (10 μM, 30 min) reduced the translocation of WT GFP-*m*SK1 in response to carbachol, S1P, or PMA (Fig. 3, *A* and *B*). The dependency on G_q_ in regulating the translocation of SK1 in response to PMA is supported by evidence demonstrating that PMA can induce the dynamic palmitoylation of Gα_q_, essential for G_q_ function (32). Confirmation that YM254890 was effective at inhibiting G_q_-mediated signaling was obtained by results showing that in MCF-7L cells loaded with the fluorescence Ca^2+^ indicator, Cal-520/AM, YM254890 (10 μM) was effective at abolishing carbachol-stimulated Ca^2+^ mobilization (Fig. S2).

**Figure 4:**
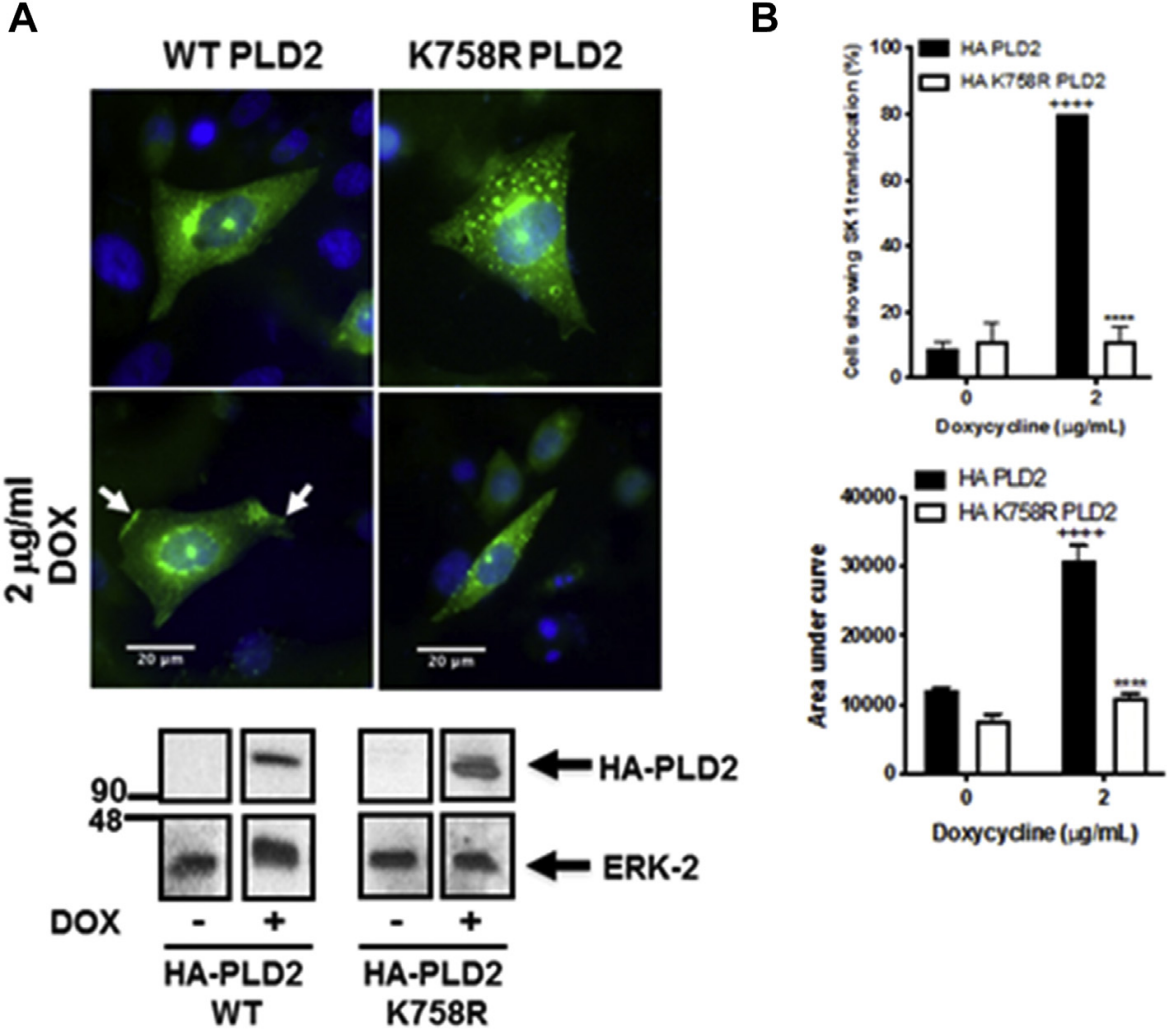
**PLD2 dependency of SK1 translocation.** WT HA-PLD2–inducible or K758R-inactive HA-PLD2–inducible Chinese-hamster ovary cells were transiently transfected with WT GFP-*m*SK1 construct and then treated with 2 μg/ml doxycycline for 24 h. *A*, photomicrographs of cells of 40× oil magnification overexpressing WT GFP-*m*SK1 detected with GFP. Representative results of three independent experiments. Also shown is a Western blot of cell lysates with the anti-HA antibody, detecting induced PLD2, and ERK-2 (loading control). *B*, the bar graphs represent the AUC of transfected WT GFP-*m*SK1 translocation (n = 5) and the percentage of cells containing translocated WT GFP-*m*SK1 (n = 3). ^++++^*p* < 0.001 doxycycline *versus* control and ^∗^^∗^^∗^^∗^*p* < 0.001 for WT PLD2 *versus* K758R-inactive PLD2 in doxycycline-treated cells (two-way ANOVA with Tukey's post hoc test). AUC, area under the curve; *m*SK1, mouse SK1; SK1, sphingosine kinase 1.

To provide additional evidence to confirm the role of PLD in regulating translocation of SK1 to the PM, we used Chinese-hamster ovary cells overexpressing doxycycline-inducible WT phospholipase D2 (PLD2) or K758R-inactive PLD2. Western blot analysis confirmed the WT and K758R PLD2 overexpression with doxycycline. Induction of WT PLD2 promoted the translocation of the WT GFP-*m*SK1. This was not the case with the K758R-inactive PLD2 mutant (Fig. 4, *A* and *B*). These results confirm that PLD2 activity is required for translocation of SK1 to the PM.

### Characterization of the monomer–dimer equilibrium

**Figure 5:**
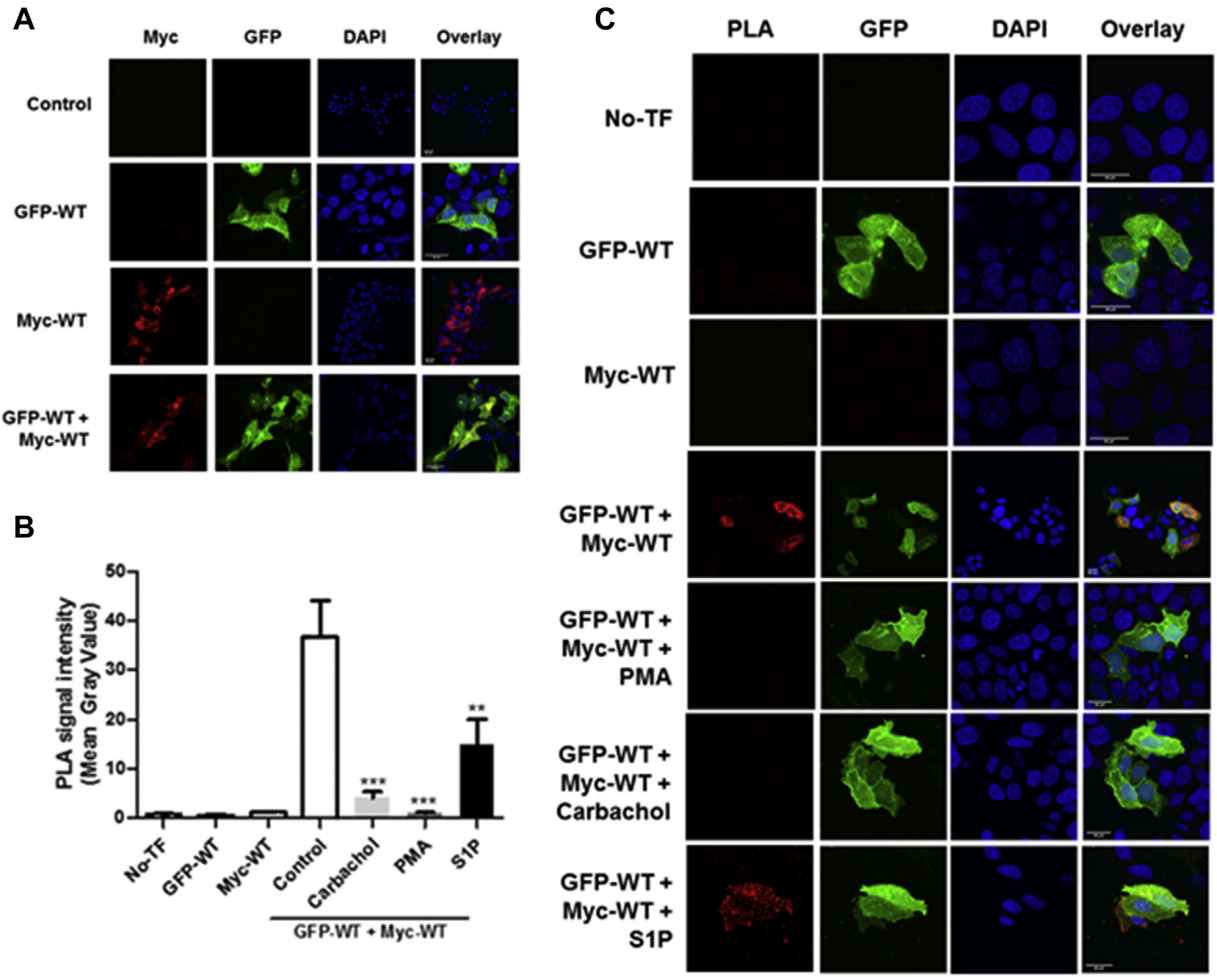
**Conditional changes in protein interaction detection between WT Myc-*m*SK1 and WT GFP-*m*SK1 using Duolink Proximity Ligation Assay (PLA).***A*, overexpression of WT Myc-*m*SK1 or WT GFP-*m*SK1 was confirmed in MCF-7L cells by fluorescence microscopy before Duolink PLA to assess protein interaction. PLA was carried out to assess differences in protein interaction between WT Myc-*m*SK1 and WT GFP-*m*SK1 (unstimulated control) and upon treatment with either S1P (5 μM) or PMA (1 μM) or carbachol (100 μM) for 10 min. Untransfected cells (No-TF) and cells expressing only WT GFP-*m*SK1 or WT Myc-*m*SK1 were used as negative controls. Cells were processed for PLA and mounted with DAPI to stain the DNA (*blue*). *B*, quantitative analysis of the cellular PLA signals was carried out. The bar graphs represent the PLA signal mean gray value (mean ± SEM) with experiments carried out in triplicate (30 cells total per treatment group shown, n = 3) ^∗^^∗^*p* < 0.01 and ^∗^^∗^^∗^*p* < 0.001 for stimulated *versus* control in WT Myc-*m*SK1/WT GFP-*m*SK1 transfected cells (one-way ANOVA with Bonferroni post hoc test). *C*, representative PLA images are shown for each treatment group. The scale bar represents 20 μm. Results are representative of three independent experiments. *m*SK1, mouse SK1; PMA, phorbol 12-myristate 13-acetate; S1P, sphingosine-1-phosphate.

To provide support for the monomer–dimer equilibrium model regulating translocation of SK1 to the PM in a ligand-specific manner, we used proximity ligation assays (PLAs) in which WT GFP-*m*SK1 and WT Myc-tagged *m*SK1 were overexpressed in MCF-7L cells. This is a ‘dimer formation’ assay, and prevention of dimer formation therefore equates with dissociation (accompanied by a reduced signal) under conditions where the dimer pre-exists. In unstimulated cells that were cotransfected with WT GFP-*m*SK1 and WT Myc-tagged *m*SK1, there was a significant PLA signal in the cytoplasm, indicating that a substantial pool of SK1 is dimeric in the cytoplasm of these cells (Fig. 5, *A*–*C*). Treatment of cells with either carbachol or PMA virtually abolished the PLA signal, thereby demonstrating that these ligands prevent formation of dimeric SK1 from monomers (Fig. 5, *B* and *C*). These findings indicate that PMA or carbachol shifts the monomer–dimer equilibrium in favor of the monomer. In contrast, a PLA signal was evident in cells treated with S1P, indicating that the dimer is still formed in cells treated with S1P, albeit less than in untreated cells (Fig. 5, *B* and *C*). Therefore, the monomer–dimer equilibrium can be influenced in a ligand-specific manner. These findings, together with data in Figure 2, indicate that the monomer translocates to lamellipodia in response to carbachol or PMA, whereas the dimer might translocate to the filopodia in response to S1P.

### Characterization of the translocation of SK1 dimerization interface mutants

**Figure 6:**
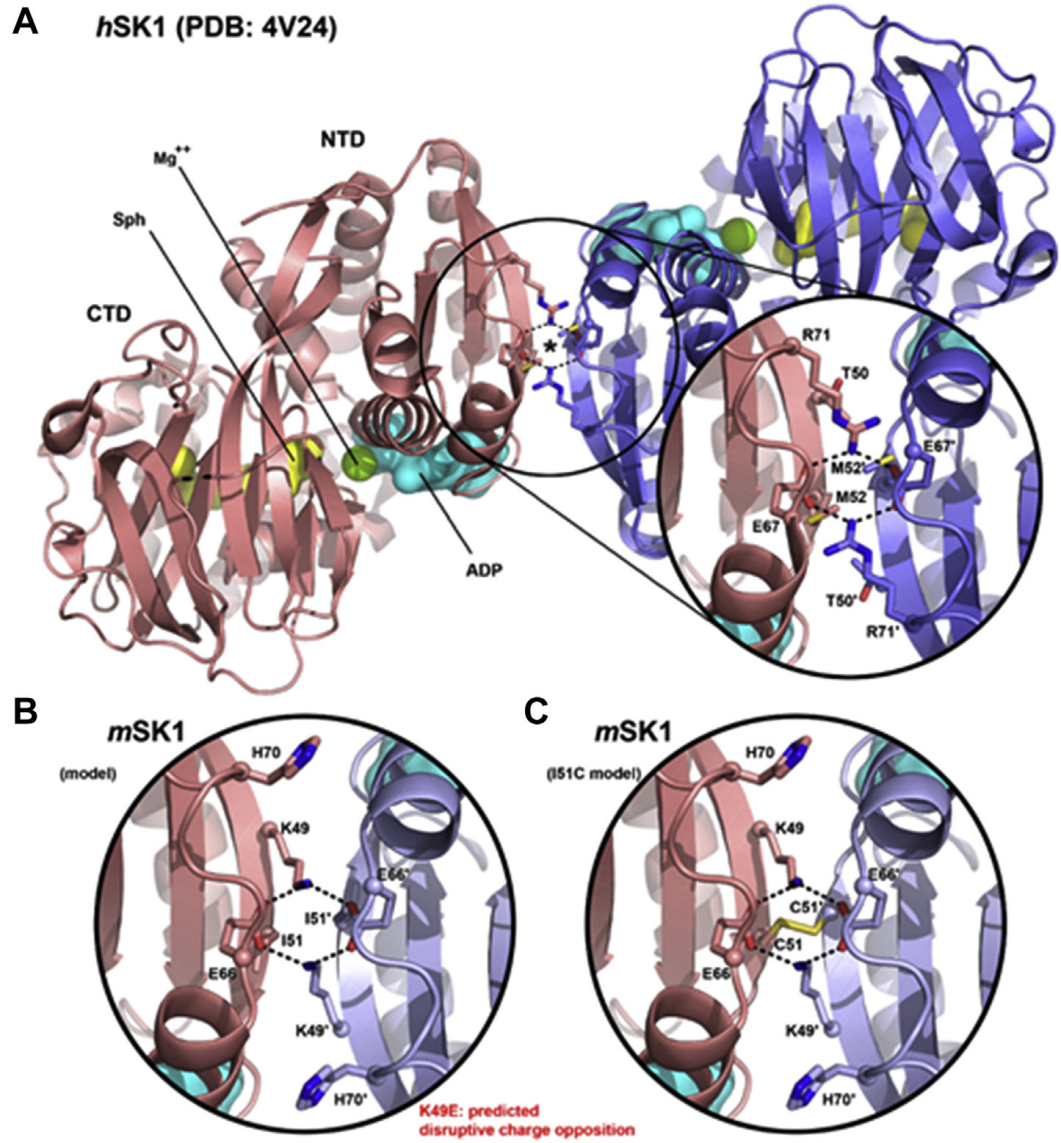
**SK1 dimerization interface and rationale for site-directed mutagenesis.***A*, crystal structures of *h*SK1 exhibit head-to-head dimerization with partial annealing of an exposed strand edge (β2) from each protomer about a C_2_-symmetry axis. A key salt bridge network comprising E67 and R71 in *h*SK1 contributes to the dimerization interface, as illustrated here from a 1.8 Å resolution structure (PDB ID: 4V24) (21) with the dimerization symmetry axis marked by an *asterisk*. The positions of bound Sph (*yellow surface*) and Mg-ADP (*green sphere/cyan surface*) are shown superimposed from separate crystal structures, 4VZB/4VZD (19). Packing of alternating hydrophobic residues (51-LML-53 in *h*SK1) along the annealed β2-strand edges also contributes significantly to the dimerization interface (M52 shown here as a *stick*). *B*, in *m*SK1, the glutamic acid of the dimerization salt bridge network is conserved (as E66), but the arginine is replaced by histidine (H70). The additional replacement of a threonine (T50) in *h*SK1 by a lysine (K49) in *m*SK1, however, suggests the existence a surrogate E66:K49:E66ʹ:K49ʹ salt bridge network to maintain the dimerization interface in *m*SK1, as illustrated here (*m*SK1 homology model). Coincident replacement of M52 in *h*SK1 with a shorter, branched side chain residue in *m*SK1 (I51, marked) creates space for the lysines and is also consistent with adoption of the postulated surrogate salt bridge network. Based on this model, we surmised that K49E charge reversal mutation would disrupt the salt bridge network (*black dotted lines*) to destabilize the dimerization interface. *C*, the *m*SK1 homology model of panel *B* indicated that the Cγ centers for the I51 and I51ʹ residues of the dimer likely lie within van der Waals contact of one another, thereby suggesting a reciprocal strategy to stabilize the dimerization interface by means of an engineered disulfide bridge (as modeled here) through the introduction of I51C substitution. *h*SK1, human SK1; *m*SK1, mouse SK1; Sph, sphingosine.

Two site-directed mutagenesis approaches were used to provide additional evidence that SK1 is subject to a monomer–dimer equilibrium and that this can determine the translocation of monomeric or dimeric SK1 to different microdomains of the PM. As there are no *m*SK1 crystal structures available at present, our strategy here was guided by analysis of the *h*SK1 crystal structures, of which there are five currently available (11). These all exhibit a common dimeric assembly of protein molecules despite the adoption of different packing arrangements for the dimers within the crystal lattices. Dimerization involves antiparallel partial annealing of an exposed β-strand from the NTD of each protomer about a C_2_ symmetry axis (Fig. 6*A*); a similar NTD–NTD dimer organization is seen almost universally across currently available crystal structures for related DAGK_cat family proteins (11). The extent of interfacial surface contact is comparatively modest, however (*ca*. 780 Å^2^ buried solvent-accessible surface area per protomer), which might be consistent with the formation of a mobile (and potentially dynamically regulated) equilibrium between monomeric and dimeric states of SK1 in a cellular context. Analysis of the dimerization interface for *h*SK1 suggests contributions from both hydrophobic surface engagement and the establishment of a highly organized polar network. There are subtle differences in the amino acid sequence for *m*SK1, but homology models (Fig. 6*B*) suggested that a lysine (K49) is likely essential for establishment of the polar interaction network in the murine enzyme through cross-dimer salt bridging. Thus, K49E charge reversal mutation is predicted to cause severe charge opposition across the dimer interface and, therefore, to impair the dimerization capacity of the enzyme. To achieve the converse effect and stabilize the dimeric state, we also sought to generate a disulfide bridge. For that purpose, we introduced an I51C mutation in the β-strand sequence (50-LIL-52, corresponding to 51-LML-53 in *h*SK1) that forms the hydrophobic core of the dimerization interface. Our models suggested that the Cγ centers of I51 in *m*SK1 likely experience contact in the dimer and that this might therefore be a reasonable site for an engineered disulfide bridge (Fig. 6*C*). Although cross-strand disulfides in certain contexts can be strained ((33) and references therein), we reasoned that a disulfide might be favorably deployed here without distorting the protein structure because the two symmetry-related β-strands diverge from one another on either side of I51. Therefore, it appears reasonable that a C51:C51ʹ disulfide bridge across the dimer symmetry axis would not attract significant penalties associated with distortion to side-by-side strand packing.

**Figure 7:**
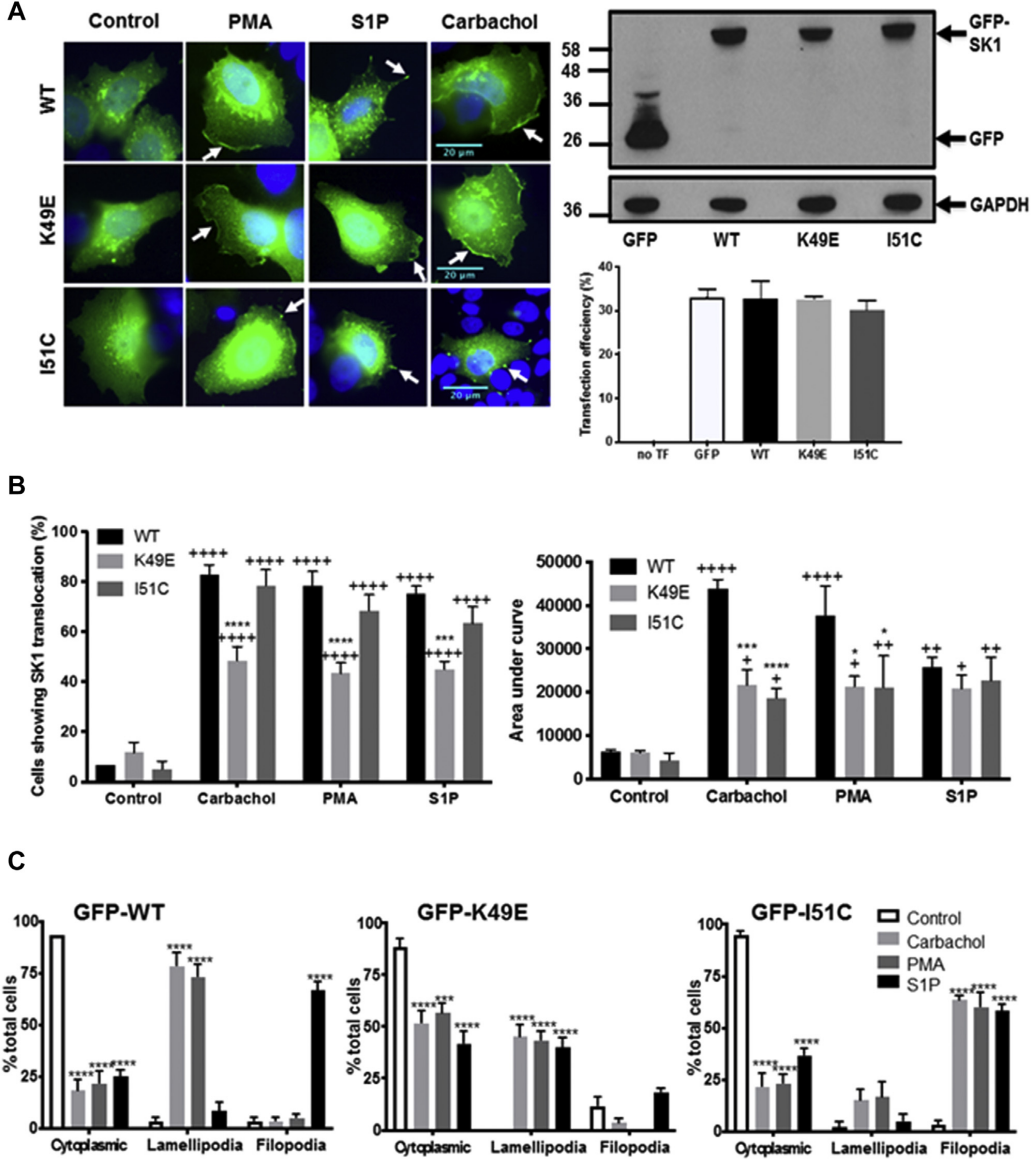
**Translocation of WT, K49E, and I51C GFP-*m*SK1 in MCF-7L cells.** MCF-7L cells overexpressing WT GFP-*mSK1* or K49E GFP-*m*SK1 or I51C GFP-*m*SK1 were treated with S1P (5 μM) or PMA (1 μM) or carbachol (100 μM) for 10 min. Cells were processed and mounted with DAPI to stain the DNA (*blue*). *A*, photomicrographs of GFP fluorescence in cells of 40× oil magnification overexpressing WT, K49E or I51C GFP-*m*SK1. Results are representative of three independent experiments. Inset is a Western blot probed with the anti-GFP antibody showing the overexpression of GFP-*m*SK1, and the bar graph shows transfection efficiency (%). No significant difference between WT *versus* mutants (one-way ANOVA with Tukey's post hoc test). *B*, the bar graphs represent the AUC (*right*) of transfected GFP-*m*SK1 (WT or mutant) translocation (n = 5) and the percentage of cells (*left*) containing translocated GFP-*m*SK1 (WT or mutant) (n = 3); ^∗^*p* < 0.05, ^∗^^∗^^∗^*p* < 0.001, and ^∗^^∗^^∗^^∗^*p* < 0.0001 for K49E or I51C mutant *versus* WT for a given stimulus; ^+^*p* < 0.05, ^++^*p* < 0.01, and ^++++^*p* < 0.0001 for stimulus *versus* respective control for transfected WT GFP-*m*SK1 or K49E or I51C mutants (two-way ANOVA with Tukey's post hoc test). *C*, bar graphs representing the percentage of cells containing GFP-*m*SK1 (WT or K49E or I51C) in the microdomains in the PM of lamellipodia and filopodia in response to stimulus (n = 4); ^∗^^∗^^∗^*p* < 0.001 and ^∗^^∗^^∗^^∗^*p* < 0.0001 for stimulus *versus* control for transfected WT GFP-*m*SK1 or K49E or I51C mutants (two-way ANOVA with Tukey's post hoc test). AUC, area under the curve; *m*SK1, mouse SK1; PMA, phorbol 12-myristate 13-acetate; S1P, sphingosine-1-phosphate.

Confirmation of the expression of K49E and I51C GFP-*m*SK1 mutants was by Western blot analysis using the anti-GFP antibody (Fig. 7*A*). The treatment of MCF-7L cells with carbachol or PMA promoted the translocation of the K49E mutant to lamellipodia, albeit this was reduced compared with WT SK1 (Fig. 7, *A*–*C*). Moreover, the treatment of MCF-7L cells with S1P also promoted the translocation of the K49E mutant to lamellipodia in the PM. This contrasts with the WT enzyme, which translocates to filopodia in response to S1P. The treatment of MCF-7L cells with S1P, PMA, or carbachol induced the translocation of the I51C mutant to filopodia, with no localization to lamellipodia (Fig. 7, *A*–*C*). Together with the redirection of the K49E mutant to lamellipodia in response to S1P, these findings suggest that S1P and carbachol do not influence translocation of SK1 by regulating the formation of lamellipodia and/or filopodia *per se*. Rather, the findings confirm that the mutants exhibit different microdomain localization in the PM, and this is governed in a ligand-specific manner.

**Figure 8:**
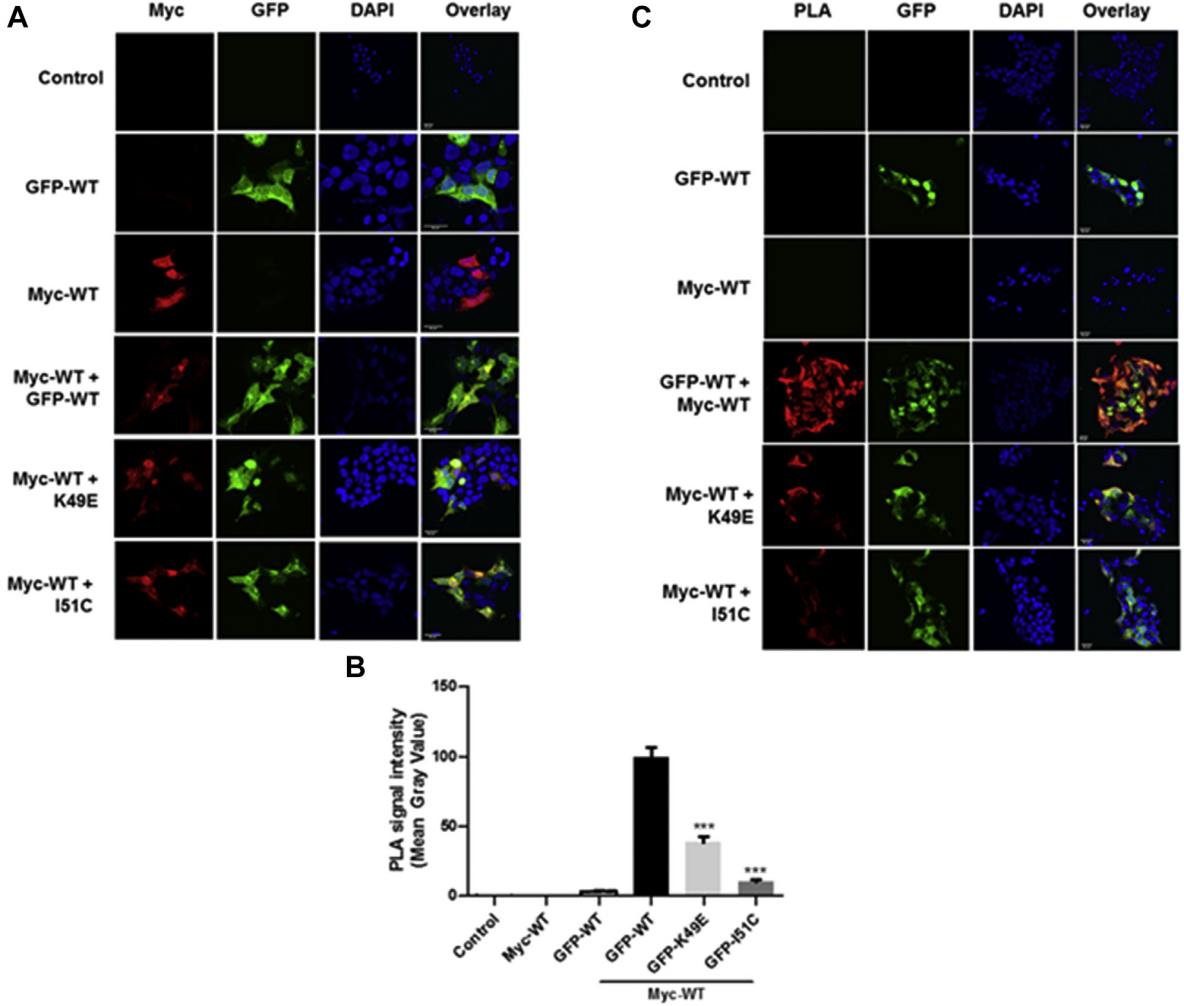
**Duolink PLA for protein interaction detection between WT Myc-*m*SK1 and WT GFP-*m*SK1, K49E GFP-*m*SK1, or I51C GFP-*m*SK1 in MCF-7L cells.***A*, MCF-7L cells overexpressing WT Myc-*m*SK1 with WT GFP-*m*SK1, K49E GFP-*m*SK1, or I51C GFP-*m*SK1 was confirmed by fluorescence microscopy before Duolink PLA to assess protein interactions. PLA was carried out to assess differences in protein interaction between WT Myc-*m*SK1 and WT GFP-*m*SK1 or K49E GFP-*m*SK1 or I51C GFP-*m*SK1 when coexpressed. Untransfected cells (control) and cells expressing only WT GFP-*m*SK1 or WT Myc-*m*SK1 were used as negative controls. Cells were processed for PLA and mounted with DAPI to stain the DNA (*blue*). *B*, quantitative analysis of the cellular PLA signals was carried out. The bar graphs represent the PLA signal mean gray value (mean ± SEM) with experiments carried out in triplicate (50 cells total per sample group shown) ^∗^^∗^^∗^*p* < 0.001 for WT Myc-*m*SK1/mutant GFP-*m*SK1 combination *versus* WT Myc-*m*SK1/WT GFP-*m*SK1 combination (one-way ANOVA with Bonferroni post hoc test). *C*, representative PLA images are shown. The scale bar represents 20 μm. *m*SK1, mouse SK1; PLA, proximity ligation assay.

We used the PLA to establish the dimerization status of the mutants. In this case, the WT GFP-*m*SK1 in the pairing with WT Myc-tagged *m*SK1 was replaced by the GFP mutants to establish whether a PLA signal can be generated. The expression levels of GFP-*m*SK1 were similar in each combination, and this was also the case for Myc-tagged *m*SK1 (Fig. 8*A*). The pairing of K49E GFP-*m*SK1 mutant with WT Myc-tagged *m*SK1 produced a low PLA signal, and this was considerably less than the PLA signal generated by the WT GFP-*m*SK1 and WT Myc-tagged *m*SK1 pairing (Fig. 8, *B* and *C*). These findings suggest that the loss of one of the two symmetry-related salt bridges involving K49 at the modeled *m*SK1 dimerization interface (Fig. 6*B*) is sufficient to substantially weaken dimerization but not to completely ablate formation of a WT Myc-tagged *m*SK1/K49E GFP-*m*SK1 heterodimer. The loss of both salt bridges and introduction of greater charge opposition is likely to weaken affinity of the protomers for each other yet further, however, such that homodimer formation with the K49E GFP-*m*SK1 mutant would be even more severely compromised, resulting in a species that is essentially monomeric when expressed in MCF-7L cells and which we show here to be localized exclusively to lamellipodia in response to carbachol or PMA challenge (Fig. 7, *A*–*C*).

Overexpression of WT Myc-tagged *m*SK1 with I51C GFP-*m*SK1 mutant produced a low PLA signal (Fig. 8, *B* and *C*). The reduced PLA signal is consistent with the I51C mutant being already locked in as a dimer and therefore unable to form a dimer with WT SK1. This is supported by the finding that the I51C mutant does not behave like a monomer as exemplified by the K49E mutant which localizes to lamellipodia, whereas the I51C mutant is localized in filopodia (Fig. 7, *A*–*C*). These findings are also in line with S1P maintaining the WT dimer and promoting its localization to filopodia, whereas PMA/carbachol promote formation of WT monomer and induce its localization to lamellipodia. Further studies concerning the I51C mutant are required.

A small amount of WT Myc-*m*SK1/I51C GFP-*m*SK1 dimer is formed, but this is considerably reduced compared with WT GFP-*m*SK1/WT Myc-*m*SK1 dimers and may reflect redox conditions controlling the integrity of disulfide bonds in the putative I51C GFP-*m*SK1/I51C GFP-*m*SK1 homodimer.

### Characterization of translocation properties for C-terminally truncated mutants (T1-T5)

**Figure 9:**
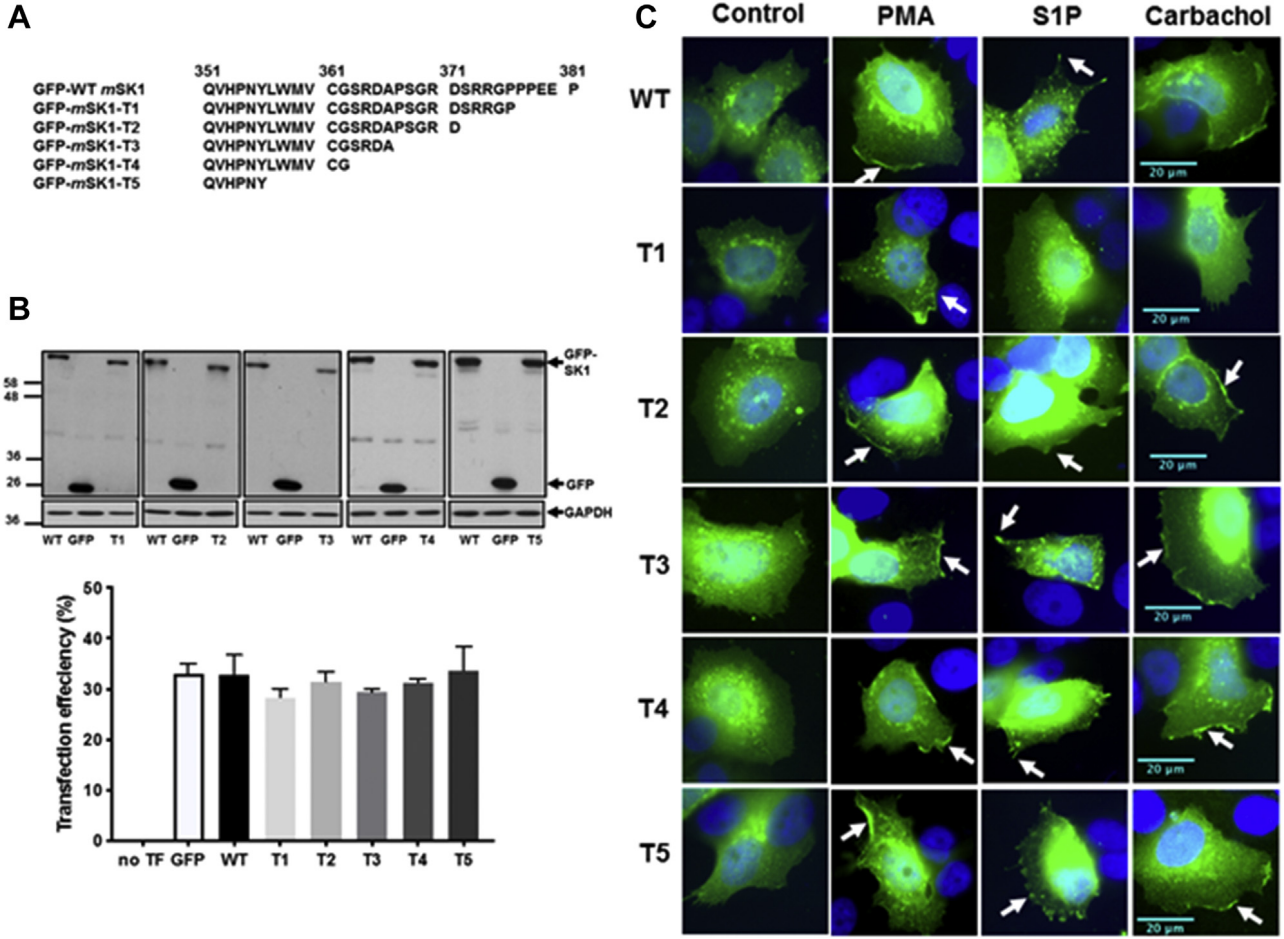

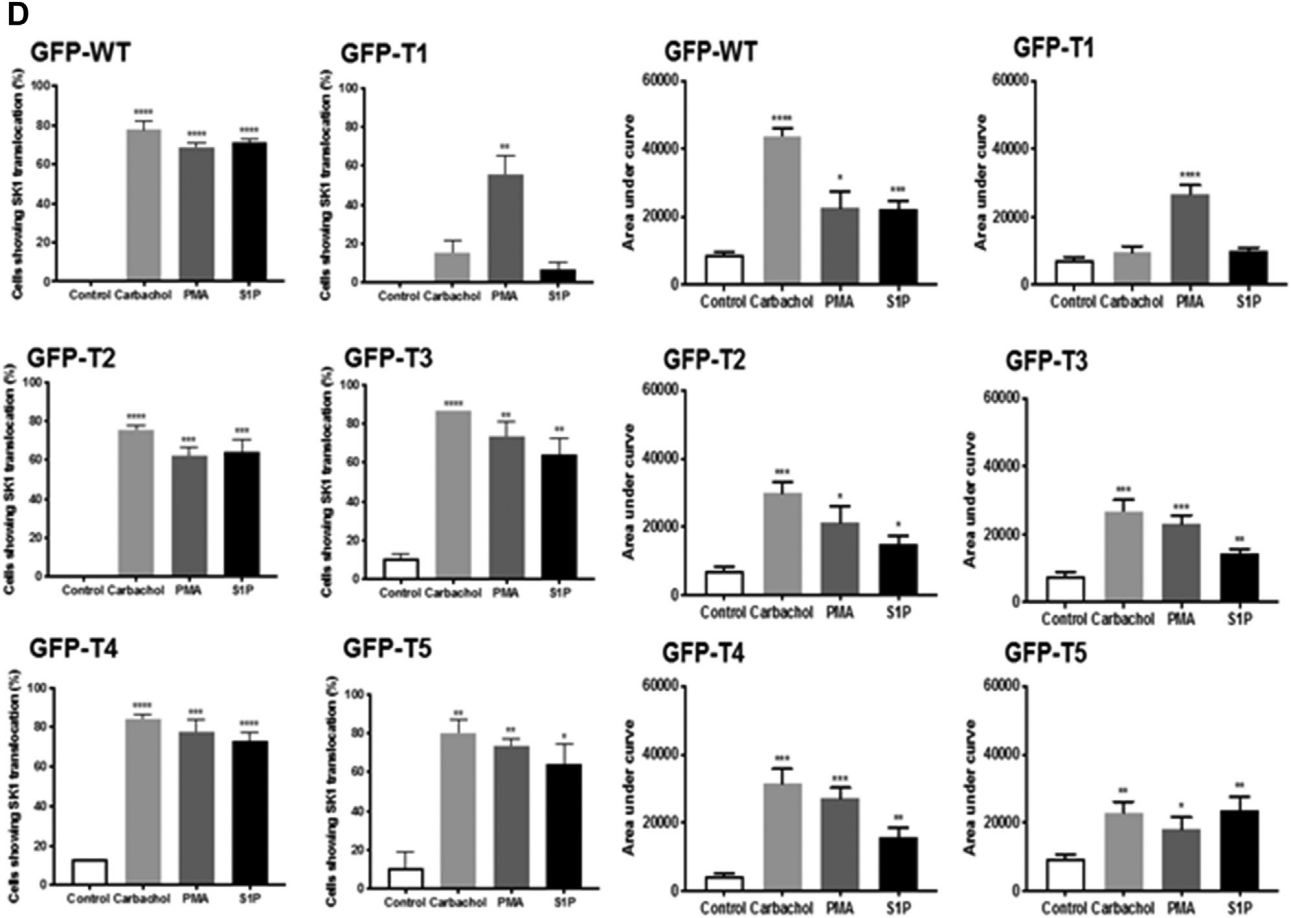
**Characterization of C-terminal tail (T1-T5) mutants.** MCF-7L cells separately overexpressing WT-GFP-*m*SK1 or each of the T1-T5 mutants were treated with S1P (5 μM) or PMA (1 μM) or carbachol (100 μM) for 10 min. Cells were processed and mounted with DAPI to stain the DNA (*blue*). *A*, schematic to show the C-terminal amino acid sequence of the T1-T5 *m*SK1 mutants. *B*, Western blot probed with the anti-GFP antibody showing the overexpression of WT and T1-T5 GFP-*m*SK1; the bar graph shows transfection efficiency. Reprobing with GAPDH is used to confirm similar protein loading. No significant difference between WT *versus* mutants (one-way ANOVA with Tukey's post hoc test). *C*, photomicrographs of GFP fluorescence in cells of 40× oil magnification separately overexpressing WT GFP-*m*SK1 or each of T1-T5 GFP-*m*SK1 mutants. Results are representative of three independent experiments. *D*, the bar graphs represent the AUC of transfected GFP-*m*SK1 (WT or T1-T5) translocation (n = 5) and the percentage of cells containing translocated GFP-*m*SK1 (WT or T1-T5) at the PM in response to stimulus (n = 3); ^∗^*p* < 0.05, ^∗^^∗^*p* < 0.01, ^∗^^∗^^∗^*p* < 0.001, and ^∗^^∗^^∗^^∗^*p* < 0.0001 for AUC for stimulus *versus* control for each of WT or T1-T5 (unpaired *t* test). AUC, area under the curve; *m*SK1, mouse SK1; PM, plasma membrane; PMA, phorbol 12-myristate 13-acetate; S1P, sphingosine-1-phosphate.

To investigate whether the C-terminal tail of SK1 is likely to have an important role in regulating its translocation to the PM, truncated forms of GFP-*m*SK1 were constructed. In these truncates (T1-T5, Fig. 9*A*) blocks of five amino acids were progressively removed from the C terminus, with the exception of T4, which had 19 amino acids removed in total. Truncates T1-T5 were separately overexpressed in MCF-7L cells, which were then stimulated with carbachol or S1P or PMA. In earlier studies, C-terminal cleavage before G364 in *h*SK1 was shown to promote binding to acidic PS-containing lipid rafts and cause constitutive activation (26). The addition of just four residues, 364-GXXX-367, restored the reduced basal activity level of *h*SK1. In *m*SK1, G364 is conserved (as G362), but the next three residues differ in identity from *h*SK1. However, Hengst *et al.* (26) had also shown that the restoration of the lower basal enzyme activity with the four-residue extension (364-GXXX-367) was independent of residue identity, suggesting that it is the peptide backbone in this region rather than specific side chains that may be important. The effect of the added sequence may potentially be due to capping at the C-terminal end of helix-α5 by the 364-GXXX-367 peptide backbone (11), but this has not been firmly established to date because G364 also represents the C-terminal limit of crystallographic structural definition in *h*SK1 at present. The G362 residue was retained in our exploratory T4 truncate but removed in the T5 construct. Confirmation of the expression of WT and T1-T5 GFP-*m*SK1 mutants was by Western blot analysis using the anti-GFP antibody (Fig. 9*B*). The translocation of the T1 mutant, lacking the last five amino acids at the C terminus (PPEEP) that are conserved in *m*SK1 and *h*SK1, was severely reduced in response to carbachol or S1P, whereas translocation in response to PMA was unaffected (Fig. 9, *C* and *D*).

**Figure 10:**
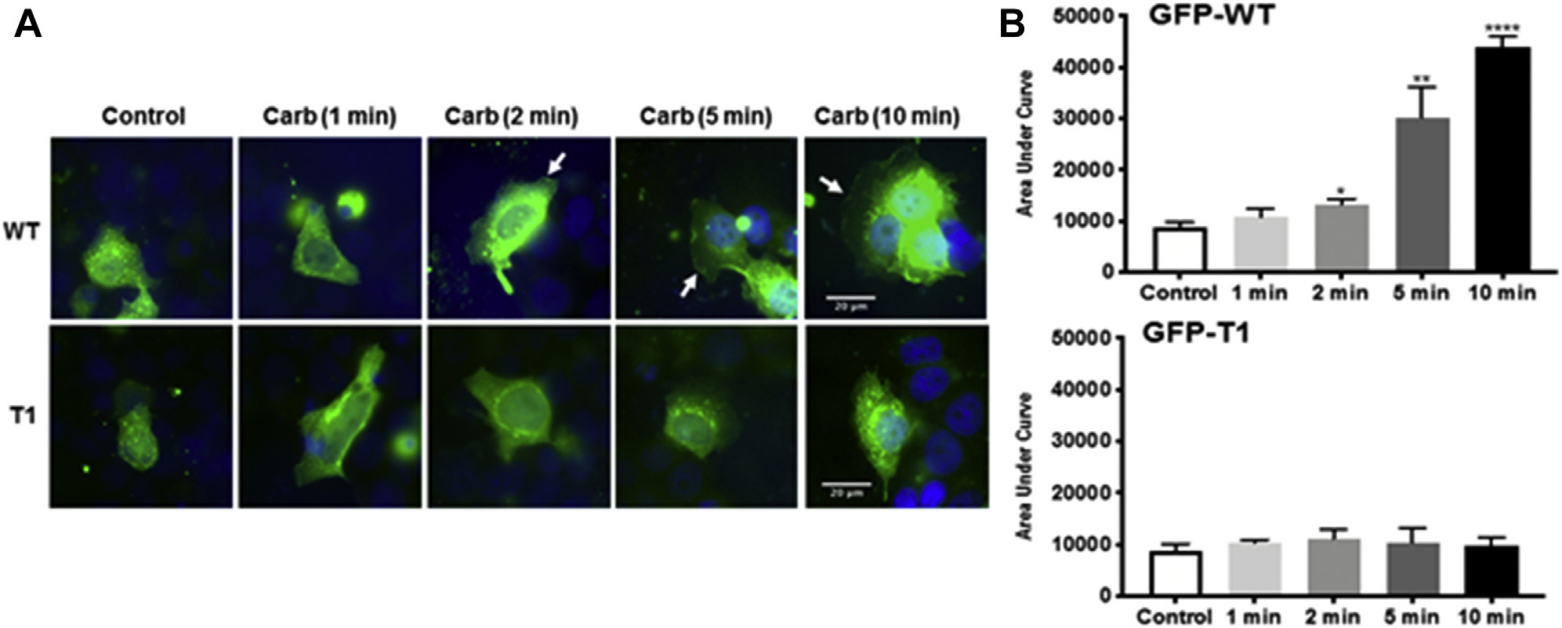
**Timecourse of SK1 translocation**. MCF-7L cells overexpressing WT GFP-*m*SK1 or T1 mutant were treated with and without carbachol (100 μM) for 1 to 10 min. Cells were processed and mounted with DAPI to stain the DNA (*blue*). *A*, photomicrographs of cells of 40× oil magnification overexpressing WT GFP-*m*SK1 and T1 GFP-*m*SK1 detected with GFP. Representative results of three independent experiments. *B*, the bar graphs represent the AUC of transfected WT GFP-*m*SK1 or T1 mutant translocation (n = 5); ^∗^*p* < 0.05, ^∗^^∗^*p* < 0.01, and ^∗^^∗^^∗^^∗^*p* < 0.0001 for treated *versus* control (unpaired *t* test). AUC, area under the curve; *m*SK1, mouse SK1; SK1, sphingosine kinase 1.

The T1 mutant failed to translocate to the PM within the 10-min stimulation period with carbachol, indicating that it does not associate and ‘fall off’ the PM at a faster rate than the WT SK1 during this time frame but simply exhibits reduced translocation to the PM (Fig. 10, *A* and *B*). In contrast, the T2-T5 mutants lacking 10 to 25 amino acids all translocated to the PM in response carbachol, S1P, or PMA and these translocations were inhibited by FIPI or YM254890 (Figs. S3, *A* and *B*, S4, *A* and *B*, S5, *A* and *B* and S6, *A* and *B*). These findings indicate the both PA and G_q_ are still required for the translocation of T2-T5 mutants, in common with the WT enzyme.

### S1P measurements

**Figure 11:**
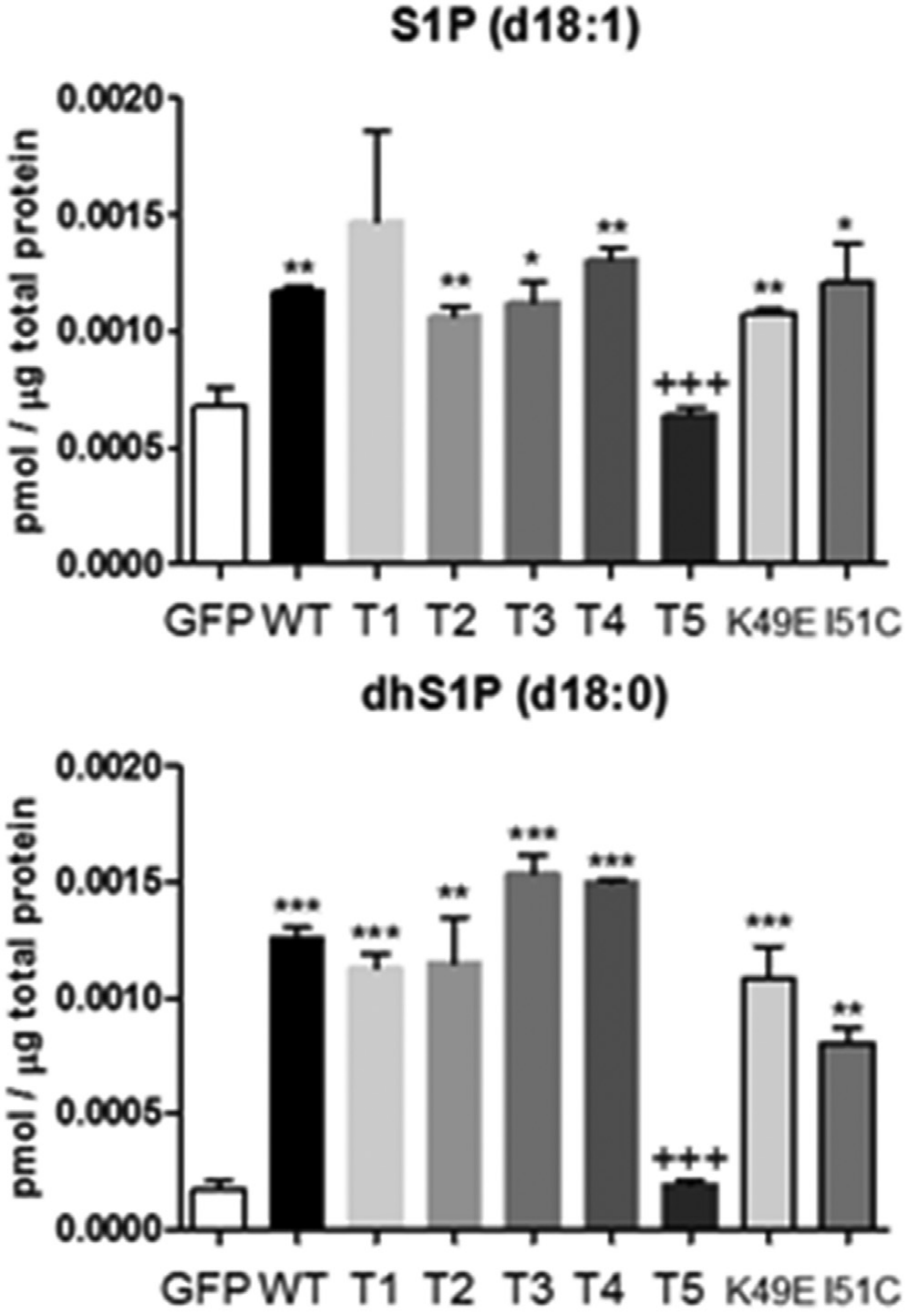
**S1P and dhS1P levels.** MCF-7L cells were transfected with WT GFP-*m*SK1 or T1-T5 GFP-*m*SK1 mutants or K49E GFP-*m*SK1 mutant or I51C GFP-*m*SK1 mutant and S1P levels measured by LC-MS/MS. ^∗^*p* < 0.05, ^∗^^∗^*p* < 0.01, and ^∗^^∗^^∗^*p* < 0.001 for SK1 (WT or mutant) transfected *versus* GFP transfected (unpaired *t* test); ^+++^*p* < 0.0001 for T5 mutant *versus* WT SK1 transfected (unpaired *t* test) for triplicate samples. *m*SK1, mouse SK1; S1P, sphingosine-1-phosphate.

To confirm the functional competence of the SK1 mutants in terms of their ability to produce S1P and to exclude an effect of mutation on activity, we measured S1P levels in MCF-7L cells overexpressing WT, K49E, I51C, and T1-T5 mutants using MS analysis. All the mutants and WT enzyme produced the same significant or approaching significant increase in S1P compared with GFP-transfected cells, with the exception of the T5 mutant, which failed to increase S1P levels above GFP-transfected cells (Fig. 11). This finding likely reflects the fact that 357-LYMVCG-362 sequence, which is present in T1-T4 but deleted in T5 (Fig. 9*A*), contributes to a critical interdomain twisted β-strand pair that is essential for the correct functional interaction of ATP-binding NTD and Sph-binding CTD halves of the enzyme (see Discussion). The helix-α5 and T-loop residues are the same between *m*SK1 and *h*SK1, consistent with evolutionary conservation of this key structural element in SK1. Similar significant increases in dihydroS1P levels were observed for WT and mutants, with the exception of T5 (Fig. 11).

## Discussion

We have shown here that monomeric or dimeric WT SK1 localizes to specific lipid microdomains in the PM dependent on the G protein–coupled receptor signaling cue (*e.g.*, carbachol, S1P). There are at least two different types of PM microdomains, notably lamellipodia and filopodia. Here we observed that carbachol-stimulated G_q_-coupled acetylcholine receptor signaling in MCF-7L cancer cells promotes translocation of monomeric WT SK1 to lamellipodia. In contrast, G_q_-coupled S1P/S1P_3_ signaling induces translocation of dimeric WT SK1 from the cytoplasm to filopodia. These data are based upon the findings that only monomeric SK1 (no dimeric SK1 is detected) is present in cells treated with carbachol or PMA, whereas dimeric SK1 is present in MCF-7L cells treated with S1P (Fig. 5), and that these forms translocate to lamellipodia or filopodia, respectively (Fig. 2). This model is supported by results using SK1 constructs engineered with mutations intended to destabilize (K49E) and stabilize (I51C) the dimerization interface. In this case, the K49E monomer localizes to lamellipodia in response to PMA and carbachol, whereas the I51C mutant moves to filopodia in response to S1P (Fig. 7).

In MCF-7L cells treated with S1P, both monomeric and dimeric WT SK1 are present, and this raises the question as to why the monomeric SK1 does not translocate to lamellipodia in addition to filopodia. This might suggest that the translocation of monomeric WT SK1 to lamellipodia is threshold dependent and that S1P does not breach this threshold, whereas carbachol and PMA do so, as supported by the PLA data obtained in this study.

Therefore, we present here a new concept that is important because one might predict that the dimeric SK1 will exhibit more avidity for the PM than the monomeric form (based on the topographically coordinated presentation of a larger number of positively charged centers and the presence of two surface-exposed hydrophobic patches for LBL-1 loops of the dimer). This might conceivably impact on the residence time that the monomer or dimer has at the PM and therefore on the magnitude of the S1P signal generated, which in turn could influence the potentially different biological effect produced in both a spatial and temporal context.

Membrane localization of SK1 is known to be sensitive to enrichment in acidic phospholipids, such as PA produced by PLD (17), and influenced by physical aspects, such as membrane curvature (12). This leaves the question of how the monomeric and dimeric forms of SK1 can sense different microdomains in the PM. This might occur because higher positive-charge density in the dimer *versus* monomer might enable the dimer to bind with more avidity to lipid microdomains in which the acidic phospholipid content is limiting for the monomer. Alternatively, the differential localization might be governed by the possibility that the dimeric assembly could be more conducive to curvature-sensitive binding to the membrane in filopodia, whereas the monomer might bind in a curvature-insensitive manner in the lamellipodia. These possibilities require further investigation.

The investigation into the role of the C-terminal tail of SK1 in regulating translocation revealed that the T1 mutant, which lacks the last five amino acids at the C terminus, is defective for translocation, whereas the T2 mutant, which lacks the last ten amino acids, is able to translocate to the PM. From these findings, it appears that amino acids 6 to 10 preceding the C terminus, which are present in T1 but not T2, may hold the enzyme in a ‘locked’ state that prevents translocation and either require the C-terminal PPEEP sequence to be present for release under conditions of G_q_ drive or require conditions that drive ERK activation for the lock to be overturned; in the latter case, the PPEEP terminus appears noncritical (*e.g.*, PMA/T1). Analysis of the available crystal structures for SK1 suggests that the position for attachment of the C-terminal tail together with the location of the R-loop, which contains the phosphorylation site for ERK, are well placed to jointly orchestrate interdomain movement that could regulate the alignment of membrane engagement determinants in the NTD and CTD.

**Figure 12:**
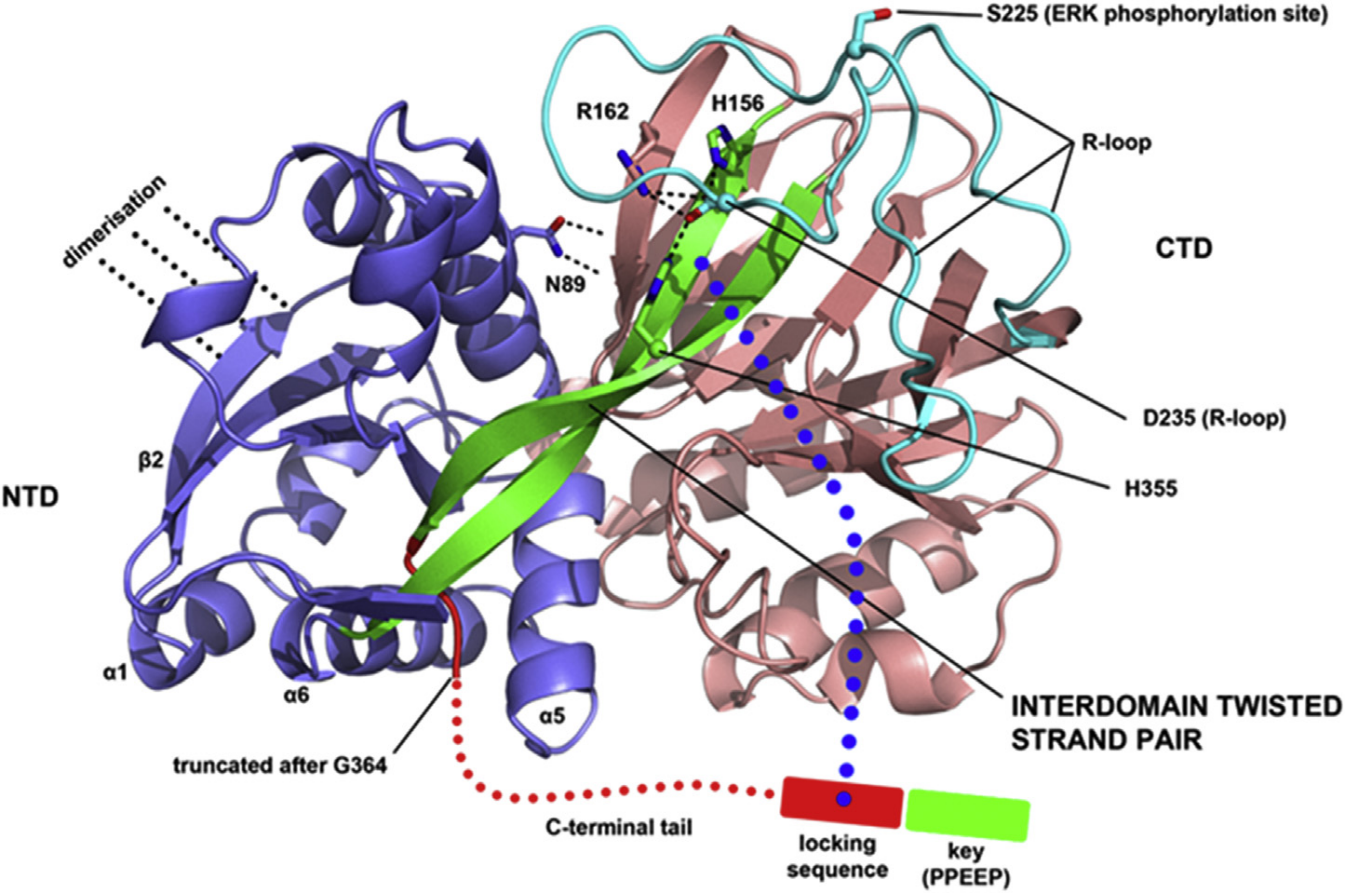
**Hypothesized structural rationale for alignment of membrane engagement determinants orchestrated by the R-loop and C-terminal tail.** SK1 exhibits a long, twisted strand pair (*green ribbon*) that acts as a ‘connecting rod’ between the NTD (*slate-blue ribbon*) and CTD (*salmon ribbon*), illustrated here from the crystal structure of *h*SK1 (PDB ID: 4L02). Flex and twist about the connecting rod is postulated to control interdomain movement and thence topographical alignment or misalignment of key membrane-interfacing determinants—K27, K29, R186, LBL-1 hydrophobic surface (obscured in this view perspective)—for anionic phospholipid-enriched membrane engagement. The protein conformation exhibited in this crystal structure, where the C-terminal tail (*red dotted line*) has been removed after G364, likely corresponds to the active state alignment of the NTD and CTD and features interdomain hydrogen bonding from N89 and engagement of connecting rod histidines (H156/H355) by R-loop D235 (labeled) to stabilize the observed conformational arrangement of domains. In cytosol, with the intact C-terminal tail, a ‘locking sequence’ (residues 6–10 preceding the C terminus in *m*SK1) is postulated to obstruct adoption of the active state conformation and maintain the NTD and CTD membrane-interfacing determinants in a misaligned state. This may involve folding of the locking sequence onto the CTD (*blue dotted line*), potentially with rotation about the twisted pair. G_q_ signaling manipulates a ‘key sequence’ (PPEEP) at the C terminus, possibly by binding of an adapter protein, to remove the influence of the locking sequence and allow adoption of the conformation with aligned membrane-interfacing determinants. R-loop phosphorylation on S225 (labeled) by ERK also drives adoption of an active state conformation in a manner that does not require the presence of the C-terminal key. In principle, this may involve a structural transition in the R-loop that overturns the hypothesized folding of the locking sequence across the CTD and forces an active-state twist on the connecting rod. Alternatively, R-loop phosphorylation might facilitate protein recruitment to stabilize the active-state conformation and overturn the locking sequence. CTD, C-terminal domain; ERK, extracellular signal–regulated kinase; *h*SK1, human SK1; LBL-1, lipid-binding loop 1; *m*SK1, mouse SK1; NTD, N-terminal domain; R-loop, regulatory loop; SK1, sphingosine kinase 1.

To date, it has not been possible to obtain SK1 crystals with the intact C-terminal tail, and the available structures are therefore truncated after G364 in *h*SK1 (corresponding to G362 in *m*SK1). The disposition of the tail residues remains to be defined, therefore. It is clear, however, that in the crystallographically observed conformation, the C-terminal tail threads through a narrow cleft between the NTD and a long twisted β-strand pair that connects the NTD to the CTD (Fig. 12, green ribbon). The ability to be able to adopt this threaded state suggests a folding mechanism that may involve initial strand alignment in a less-twisted state followed by interdomain rotation. Thus, we cannot at present rule out the possibility that translocation between the cytosol and PM may involve significant interdomain rotational movements about the twisted pair. A potential explanation for the influence of the C-terminal tail on translocation in our study, then, may be that the putative locking sequence (amino acids 6–10 preceding the C-terminus) folds across the CTD to actively maintain a conformational arrangement for the two domains that keeps the membrane interfacing determinants out of alignment for coordinated engagement of the membrane. Binding of a protein partner to the terminal acidic polyproline sequence (PPEEP) under conditions of G_q_ drive may displace the C-terminal tail from its locking position to facilitate the particular interdomain arrangement with coordinated NTD and CTD membrane-binding determinants. In principle, phosphorylation of S225 under conditions of PMA drive might elicit a structural transition in the regulatory R-loop (Fig. 12, cyan ribbon) that displaces the C-terminal tail without the need for sequestration of the PPEEP C terminus. Controlled phasing of membrane interfacing determinants in the NTD and CTD by these mechanisms might operate in both the monomer and the dimer to regulate translocation to the PM but with the dimer providing an extended interface that may influence affinity and sensitivity to membrane curvature. Further work is required to establish a detailed structural basis for the role of the C-terminal tail in regulating membrane localization.

We conclude that the C-terminal tail of SK1 can regulate the translocation of SK1 to different microdomains in the PM in a ligand-specific manner that is affected by the monomer/dimer status of the enzyme. The precise mechanistic basis for perturbation of the monomer/dimer equilibrium requires further investigation. However, we propose that this differential localization of the monomer *versus* dimer likely increases the repertoire of functions and endows the S1P that is formed, with spatial and temporal pleiotropic signaling properties. This may provide new options for treating disease by blocking the C-terminal tail function of SK1 to eliminate translocation and activation of the enzyme. In addition, the existence of a monomer–dimer equilibrium might be used to inform on therapeutic targeting strategies in cases where, for example, the dimeric SK1 might drive disease pathology, whereas the monomeric form does not or *vice versa*.

## Experimental procedures

### Materials

Cell culture reagents including high-glucose Dulbecco's modified Eagle's medium (DMEM), antibiotics and LipofectAMINE 2000 were purchased from Invitrogen. S1P was from Avanti Polar Lipids, YM254890 from Caltag Medsystems, FIPI from Cayman Chemical Company, and PD98059 from Sigma-Aldrich Company Ltd. Antibodies used were anti-GAPDH (sc-47724), anti-GFP (sc-9996), and anti-phosphoERK1/2 (sc-7383) from Santa Cruz (through Insight Biotechnology Ltd), anti-ERK2 (#610104) from BD Biosciences, and anti-HA tag antibody (H3663) and anti-actin (A2066) were from Sigma-Aldrich Company Ltd. Anti-fascin (#54545), anti-paxillin (#2542), and anti-cortactin (#3503) antibodies were purchased from Cell Signaling Technology. The anti-SK1 antibody was custom-synthesized by Abgent using the antigens H_2_N-CPRGGKGKALQLFRSH-CONH_2_ and H_2_N-CPRGGKGKALQLFRSH-CONH_2_ (34). VECTASHIELD with 4′,6-diamidino-2-phenylindole (DAPI) was from Vector Laboratories, UK. Plasmid DNA used in this study was purified using endotoxin-free plasmid preparation kits and their sequences validated by Sanger sequencing (GATC). Primers were synthesized by Integrated DNA Technologies, BVBA.

### Cell culture

MCF-7L cells were maintained in a humidified incubator at 37 °C, with CO_2_ (5% (v/v)) in DMEM supplemented with fetal calf serum (FCS) (10% (v/v)), penicillin (100 U/ml), and streptomycin (100 μg/ml) (complete DMEM). PLD2-inducible Chinese-hamster ovary cells were maintained in Ham’s F12 medium, respectively, supplemented with 10% (v/v) FCS (and penicillin (100 U/ml)/streptomycin (100 μg/ml)). In all cases, cells were deprived of serum for 24 h before experimentation.

### Molecular biology

Truncations and site-directed mutagenesis of a construct encoding GFP-tagged WT *m*SK1 (WT GFP-*m*SK1) was by PCR using pFuUltra II fusion Hot start high-fidelity DNA polymerase (Agilent Technologies LDA UK Limited) (95 °C for 2 min to activate the polymerase, followed by thermal cycling of 95 °C for 20 s, 60 °C for 20 s, 72 °C for 105 s, 20 cycles) and specific primers. Truncations were of 5, 10, 15, 19, or 25 C-terminal amino acids (termed GFP-*m*SK1-T1 to T5, respectively).

Primer sequences (5’-3’)

GFP-*m*SK1-T1,

Forward: ACTCCCGGCGGGGGCCATAACCAGAAGAACCATAATC

GFP-*m*SK1-T1,

Reverse: GATTATGGTTCTTCTGGTTATGGCCCCCGCCGGGAGT

GFP-*m*SK1-T2,

Forward: CATCCGGCCGGGACTAACGGCGGGGGCCACCT

GFP-*m*SK1-T2,

Reverse: AGGTGGCCCCCGCCGTTAGTCCCGGCCGGATG

GFP-*m*SK1-T3,

Forward: TGGCAGCAGAGATGCCTAATCCGGCCGGGACTCCCG

GFP-*m*SK1-T3,

Reverse: AGTCCCGGCCGGATTAGGCATCTCTGCTGCCA

GFP-*m*SK1-T4,

Forward: CCTTTGGATGGTCTGTGGCTGAAGAGATGCCCCATCCGG

GFP-*m*SK1-T4,

Reverse: CCGGATGGGGCATCTCTTCAGCCACAGACCATCCAAAGG

GFP-*m*SK1-T5,

Forward: CAAGTGCACCCAAACTACTAATGGATGGTCTGTGGCAG

GFP-*m*SK1-T5,

Reverse: CTGCCACAGACCATCCATTAGTAGTTTGGGTGCACTTG

GFP-*m*SK1-K49E,

Forward: GCAGAGATAACCTTTGAACTGATACTCACCGAAC

GFP-*m*SK1-K49E,

Reverse: GTTCGGTGAGTATCAGTTCAAAGGTTATCTCTGC

GFP-*m*SK1-I51C,

Forward: GAGATAACCTTTAAACTGTGCCTCACCGAACGGAAGAACC

GFP-*m*SK1-I51C,

Reverse: GGTTCTTCCGTTCGGTGAGGCACAGTTTAAAGGTTATCTC

### Transfections

MCF-7L cells, plated for 24 h or until 70% confluence (and on coverslips, where indicated), were transfected with of the DNA (1 μg), as indicated in legends, using LipofectAMINE 2000 according to the manufacturer’s instructions. The transfection mixture was added dropwise to cells and incubated for 24 h. Cells were further incubated in serum-free DMEM for 24 h before treatment (see figure legends).

### Treatments

Cells were treated with vehicle, carbachol (100 μM final), S1P (5 μM), or PMA (1 μM) for 10 min. Where indicated (see figure legends), cells were preincubated with PD98059 (50 μM) (inhibitor of MEK1 and MAP kinase cascade) for 1 h, YM254890 (G_q_ inhibitor) (10 μM) for 30 min, or FIPI (PLD1/2 inhibitor) (100 nM) for 1 h.

### Western blotting

Cells were harvested by removal of the media and addition of Laemmli sample buffer (200 μl; SDS (0.5% (w/v)), Tris HCl (125 mM, pH 6.7), EDTA (1.25 mM), sodium pyrophosphate (0.5 mM), DTT (50 mM), bromophenol blue (0.06% (w/v)), and glycerol (12.5% (v/v))) before homogenization using a 23-gauge hypodermic needle and syringe. Samples were analyzed by SDS-PAGE in parallel with prestained molecular weight markers and Western-blotted for proteins of interest. Similar protein loading between samples was determined by reprobing for either ERK2 or GAPDH.

### Immunofluorescence microscopy

Cells grown on 13-mm autoclaved glass coverslips were transfected, if required, as above and incubated in serum-free DMEM for 24 h before treatment (see figure legends). Cells were fixed with formaldehyde (3.7% (v/v) in PBS) for 15 min and permeabilized using Triton X-100 (0.1% (v/v) in PBS) for 2 min. For the detection of transfected forms of GFP-*m*SK1, the coverslips were washed briefly with PBS and mounted on glass slides using VECTASHIELD hard-set anti-fade mounting medium with DAPI. For the detection of endogenous proteins, the coverslips were incubated with blocking buffer (FCS (5% (v/v)) and BSA (1% (w/v)) in PBS) for 30 min before incubation with the primary antibody (1:50) in the blocking buffer overnight at 4 °C. After briefly washing in PBS, coverslips were incubated with FITC- or tetramethylrhodamine-isothiocyanate-conjugated anti-mouse IgG or anti-rabbit IgG secondary antibody (1:50) in the blocking solution for 1 h at room temperature (RT). Coverslips were washed in PBS before mounting on glass slides using VECTASHIELD with DAPI. Samples were imaged for DAPI and GFP (FITC settings) using an epifluorescence upright microscope (Nikon, Eclipse E600) with 40× oil lens accompanied with WinFluor imaging software.

### Quantification of GFP-*m*SK1 translocation

For quantification of the percentage of cells displaying localization of lamellipodia or filopodia/foci of GFP-SK1, immunofluorescence images of 15 cells (randomly chosen) were acquired from three or more experiments. These were assessed (nonblinded) for evidence of GFP-*m*SK1 translocation to the PM and whether this was predominantly lamellipodia or localized to filopodia/foci. ImageJ (Scion Corporation) analysis of raw images acquired for GFP alone (no DAPI signal) was also used to quantify PM translocated GFP-*m*SK1. The segmented line tool was used to measure the pixel intensity (on a scale of 0–256 (black–white), corresponding to increasing amounts of GFP-*m*SK1) for a distance of 500 pixels at the PM. Data from five cells were combined in series (distance of 2500 pixels). The average pixel intensity (2500 pixels) of five control cells was subtracted as background fluorescence for each experiment. All images were of similar brightness and contrast. The AUC of a point-to-point plot was calculated as a quantitative measure of GFP-*m*SK1 translocation for a given treatment or SK1 mutant.

### PLA

Duolink *In situ* Orange proximity ligation assays (DUO92102, Sigma-Aldrich) were carried out in MCF-7L cells transfected with WT GFP-*m*SK1 or K49E GFP-*m*SK1 or I51C GFP-*m*SK1 mutant constructs and/or Myc-tagged *m*SK1. MCF-7L cells were seeded in 12-well plates containing 13-mm-diameter glass coverslips before transfection as previously detailed. Interaction was first determined in unstimulated cotransfected cells and upon treatment with carbachol (100 μM), S1P (5 μM), and PMA (1 μM) for 10 min. Further experiments were carried out to assess interaction between Myc-*m*SK1 (WT) and GFP-SK1 mutants (K49E and I51C). Cell fixation, permeabilization, and blocking were carried as detailed in the Immunofluorescence microscopy section. Coverslips were incubated with rabbit anti-GFP (Clontech, Takara) and mouse anti-Myc-Tag primary antibodies overnight (1:500). PLAs were performed as per manufacturer’s instructions using the Duolink *In Situ* (orange) range of detection reagents, wash buffers, and PLA probe kits. All incubation steps were carried out in a humidity chamber at 37 °C. After primary antibody incubation, cells were washed twice in Duolink wash buffer A for 5 min and then incubated in rabbit PLUS and mouse MINUS Duolink PLA probes used at a 1:5 dilution in the Duolink Antibody Diluent at 37 °C for 1 h. After incubation, cells were washed twice in Duolink wash buffer A for 5 min and then incubated in the Duolink ligation buffer (1:5) containing 1 U ligase at 37 °C for 30 min. Coverslips were washed twice in buffer A for 5 min and incubated in the Duolink amplification buffer (1:5) containing 0.5 U polymerase at 37 °C for 100 min (photosensitive reaction). Cells were washed in Duolink wash buffer B for 10 min at RT followed with a final wash in 0.01× buffer B for 1 min. Cells were counterstained with DAPI (500 nM) for 5 min and washed three times with PBS, and the coverslips were mounted on to glass microscope slides with Mowiol. PLA-positive cells were visualized using a Leica TCS SP8 confocal microscope at magnification of 63× with an oil-immersion lens (PLA Orange wavelengths λ_ex_ 554 nm and λ_em_ 576 nm). GFP-transfected cells were used as a hallmark of successful transfection (FITC settings). PLA experiments were carried out blinded. Image acquisition was carried out using Leica LAS software, with images processed *via* ImageJ (Scion Corporation). Cellular PLA signal intensities were measured for each positive cell, with cell populations averaged for each treatment group (50 cells/treatment) and presented as the mean ± SEM with analysis carried out using GraphPad Prism 9. Indirect immunofluorescence was carried out in parallel to PLA to demonstrate successful cotransfection and expression of target proteins of interest as described previously.

### Lipidomics

#### Single-phase lipid extraction and derivatization

A single-phase butanol:methanol (1:1, BuMe) lipid-extraction protocol was used to extract S1P from cells. Cell pellets were mixed with 400 μl of BuMe and 200 μl of internal standard solution containing S1P d18:1 ^13^C_2_D_2_ in BuMe. Samples were mixed with a vortex for 10 s and sonicated with ice in an ultrasonic bath for 30 min. Samples were then centrifuged at 14,000*g* for 10 min at RT. A volume of 250 μl of the supernatant from each sample was transferred to Eppendorf tubes, dried completely in a speed-vac, and resuspended in 90 μl of BuMe. S1P species were derivatized as described (35). A volume of 30-μl trimethylsilyl-diazomethane was added to the resuspended extracts, which were incubated in a thermomixer at 1000 rpm for 20 min at RT. To stop the reaction, 1 μl of glacial acetic acid was added to each sample. Samples were mixed with a vortex mixer and centrifuged at 16,000*g* at RT. The supernatants were transferred to the MS vial for LC-MS analysis.

#### S1P analysis

A chromatographic separation was performed on a Waters ACQUITY UPLC BEH HILIC (130 Å, 2.1× 100 mm, 1.7 μm) column, thermostatted at 60 °C in an Agilent 1290 UHPLC system. The flow rate was 400 μl min^−1^ with mobile phase A composed of acetonitrile and 25 mM ammonium formate buffer (50:50, v/v) and mobile phase B composed of acetonitrile and 25 mM ammonium formate buffer (95:5, v/v). Mobile phases A and B were mixed to create the following gradient: 99.90% B at 0 min to 40% B at 5.00 min and 10% B at 6.50 min and reequilibrated at 99.90% B from 6.60 min to 9.60 min. The total run time was 9.60 min. An Agilent 6495 QQQ was used for targeted measurements. The Agilent Jet Stream ESI source parameters for the MS were dry gas temperature and flow of 200 °C and 12 l min^−1^, respectively, nebulizer pressure 25 psi, sheath gas temperature and flow were set to 400 °C and 12 l min^−1^, respectively, capillary voltage and nozzle voltage set to 3500 V and 500 V, respectively, and the delta electron multiplier voltage of 200 V. Positive high/low pressure radio frequency was set to 200/110.

#### *MS data analysis*

The acquired MS data were analyzed using Agilent MassHunter software, version B.08.00. The signal-to-noise ratios were calculated using the raw peak areas in the study samples and processed blanks. Lipids that had signal-to-noise ratios <10 and a covariance >30% (calculated from a pooled quality control analyzed every five samples) were discarded. Internal standards were used to normalize the raw peak areas using corresponding quantifier/qualifier transitions. All species were normalized to S1P d18:1 ^13^C_2_D_2_. The values after normalization to internal standard were further normalized to the protein amount. The MS was operated in positive ionization mode and a multiple reaction monitoring method was set up for the analysis. For each S1P, two product ions at *m/z* 60.08 (quantifier) and *m/z* 113 (qualifier) generated from the trimethylsilyl-derivatized tetramethylated form of S1P were monitored.

### Calcium measurements

Intracellular Ca^2+^ was monitored using the Ca^2+^-sensitive fluorescent indicator, Cal-520 (36, 37). For this, MCF-7L breast cancer cells were plated in ibidi μ-Slide 8-well culture dishes and left to adhere for 24 h (37 °C, 5% CO_2_). After 24 h, the media was replaced with serum-free media for 24 h. Cells in the ibidi chamber were incubated in DMEM containing Cal-520-AM (5 μM with 0.04% Pluronic F-127 and 0.26% dimethylsulphoxide for 30 min at 37 °C in the dark. Ca^2+^ imaging was performed using an inverted fluorescence microscope (TE2000U; Nikon) equipped with a 40× objective (oil immersion, 1.3 numerical aperture; Nikon S Fluor). Fluorescence emission was recorded at 10 Hz using a large-format (1024 × 1024 13 μm pixels) electron multiplying charge-coupled device camera (iXon 888; Andor). Cal-520 AM was excited with 488-nm wide-field epifluorescence illumination provided by a monochromator (Photon Technology International/Horiba UK, Ltd). Ca^2+^ activity was recorded at RT for 5 min. A stable baseline was established for the first 60 s of the recording before the addition of carbachol (100 μM). To examine the effects of the selective G_αq/11_ inhibitor YM254890 (10 μM), MCF-7L cells were incubated in YM254890 for 30 min at 37 °C before the addition of carbachol. Temporal Ca^2+^ signals were extracted from the raw fluorescence intensity image stacks, using 30-pixel (∼4 μm) diameter circular regions of interest manually positioned at the center of each cell. Ca^2+^ signals were analyzed using a custom Python-based analysis suite as described previously (36, 37). The average initial Ca^2+^ peak response evoked by carbachol in the absence and presence of YM254890 was analyzed in GraphPad Prism, v7.01 (GraphPad).

### Statistics

Data are presented as the mean ± SEM and were statistically analyzed using GraphPad Prism 7 using one or two-way ANOVA and Tukey's multiple comparison test or Bonferroni’s post hoc test or by unpaired two-tailed *t* test.

### Pearson correlation coefficients

Colocalization of proteins of interest was performed using the JACoP plugin for ImageJ to measure Pearson’s correlation coefficient (plotted intensity of individual pixels of FITC *versus* tetramethylrhodamine-isothiocyanate for each sample (cytofluorogram)), and an r value was calculated and graphically presented.

## Data availability

All data are contained within the article.

## Supporting information

This article contains supporting information.

## Conflict of interest

The authors declare that they have no conflicts of interest with the contents of this article.
