# Supplementary material for: A new model for regulation of sphingosine kinase 1 translocation to the plasma membrane in breast cancer cells
Source: J Biol Chem. 2021 Apr 16;296:100674. doi: 10.1016/j.jbc.2021.100674 (PMC8135045; doi:10.1016/j.jbc.2021.100674)
Supplement: Supplemental Figures S1–S6 [file mmc1.pdf]

A new model for regulation of sphingosine kinase 1 translocation to the plasma membrane in breast cancer cells

Ryan D.R. Brown[1], Benjamin Veerman[1], Jeongah Oh[2], Rothwelle J. Tate[1], Federico Torta[2],

Margaret R. Cunningham[1], David R. Adams[3], Susan Pyne[1] and Nigel J. Pyne[1*]

Supplementary Figures

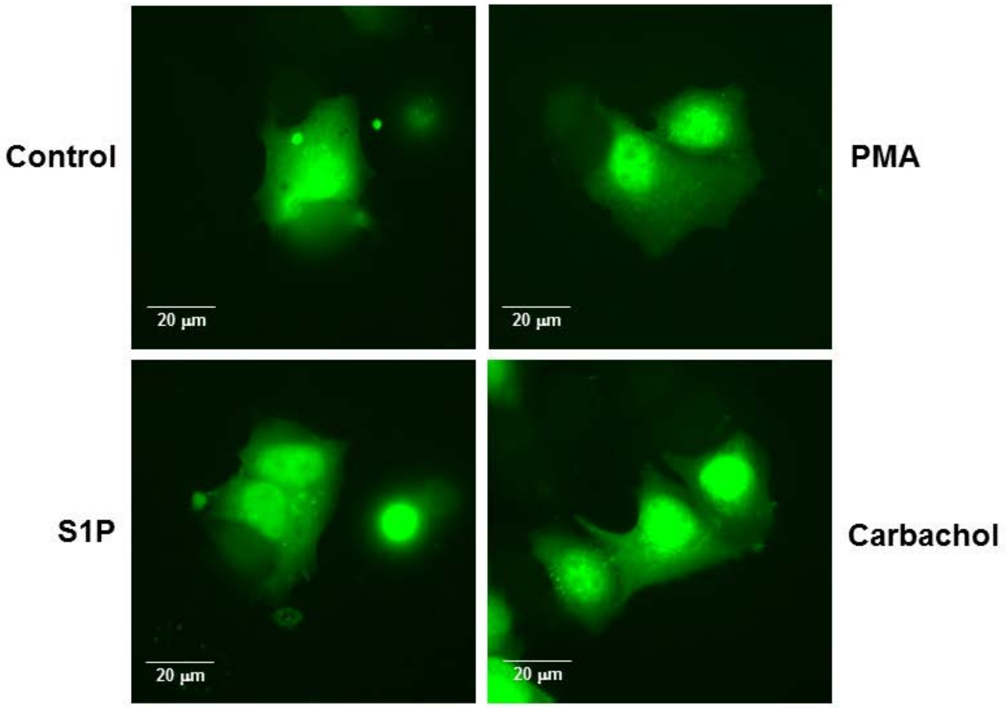

Fig. S1 Lack of effect of ligand stimulation on the redistribution of GFP in MCF-7L cells. MCF-7L cells over-expressing GFP were treated with either S1P (5 μM) or PMA (1 μM) or carbachol (100 μM) for 10 min. 40x oil magnification photomicrographs of GFP fluorescence in cells over-expressing GFP. Results are representative of 3 independent experiments.

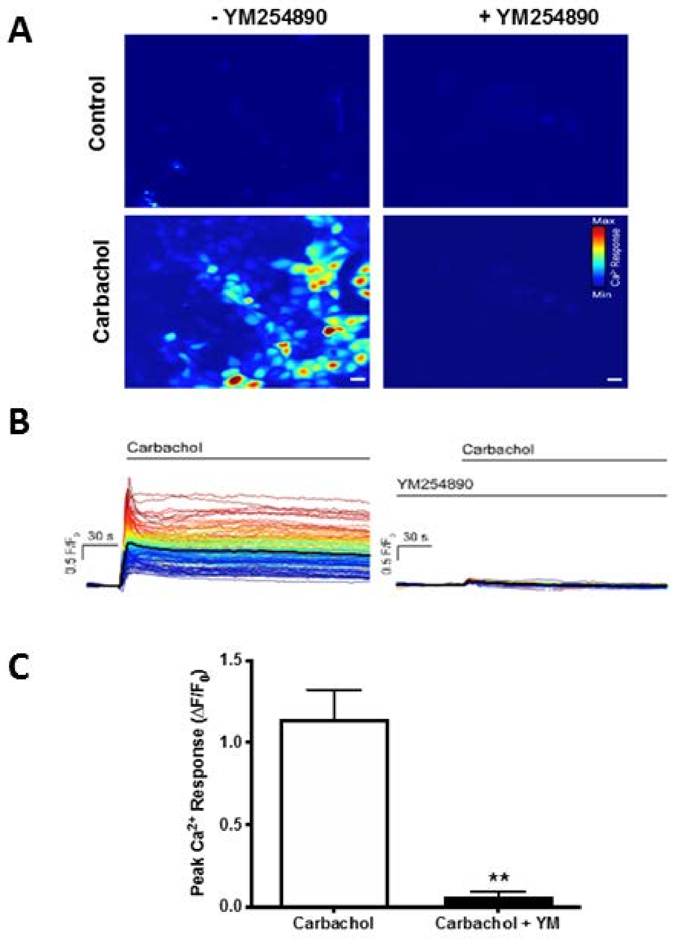

Fig. S2 Validation of the effect of the $G_{\alpha q/11}$ protein inhibitor YM254890 on $G_{\alpha q}$-mediated signalling. (A) Representative fluorescence image showing MCF-7L cells loaded with the fluorescence Ca^{2+} indicator, Cal-520/AM. The left panels shows basal Ca^{2+} and after stimulation with 100 µM carbachol; the right panels show basal and stimulated in the presence of the $G_{\alpha q/11}$ protein inhibitor YM254890 (10 µM). **(B)** Pseudo-coloured $\Delta F/F_0$ maximum intensity projection of the same field of view as shown in **(A)**. Overlaid Ca^{2+} signals from all individual cells in the field of view. Colours were assigned to traces based on the amplitude of the initial response (red indicates the highest amplitude and blue the lowest amplitude). The black line represents the mean response. **(C)** Summary data illustrating average peak response to carbachol before and after incubation with 10 µM YM254890 (n=3 for each). ** $p<0.01$ for carbachol vs carbachol with YM254890 (unpaired t test). Scale bars = 20 µm.

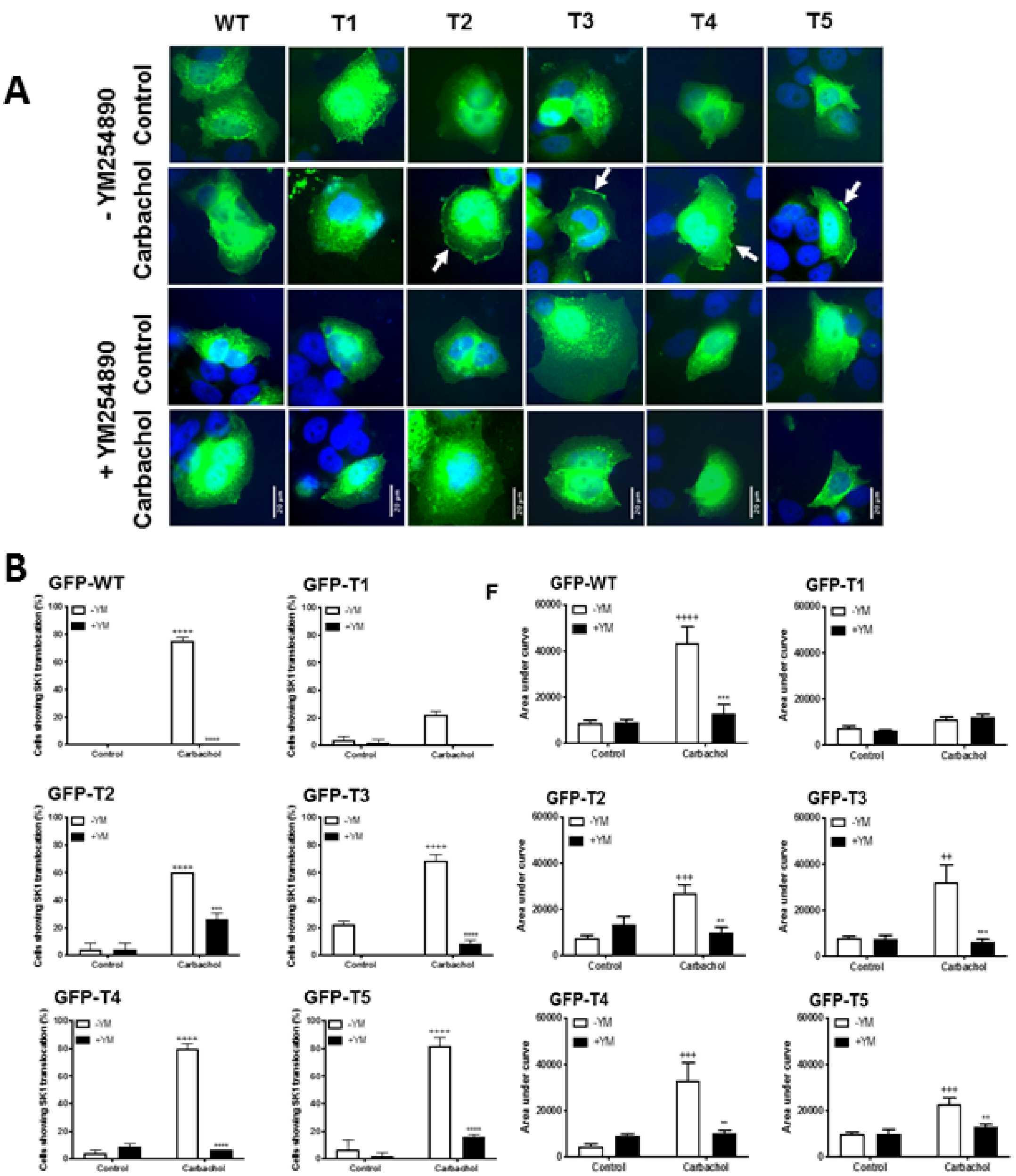

Fig. S3 Sensitivity of T1-T5 mutants to the G_q inhibitor YM254890 in response to carbachol. MCF-7L cells over-expressing WT-GFP-SK1 or T1-T5 mutants were treated with YM254890 (10 µM, 30 min) prior to carbachol (100 µM) for 10 min. Cells were processed and mounted with DAPI to stain DNA (blue). **(A)** 40x oil magnification photomicrographs of cells over-expressing WT GFP-*m*SK1 and T1-T5 GFP-*m*SK1 detected with GFP. Representative results of 3 independent experiments. **(B)** The bar graphs represent the AUC of transfected GFP-*m*SK1 (WT or T1-T5) translocation (n=5) and the % cells containing GFP-*m*SK1 (WT or T1-T5) in response to carbachol (n=3); ** p<0.01, *** p<0.001 and **** p<0.0001 for carbachol vs carbachol with YM254890 and for each of WT GFP-*m*SK1 and T1-T5; $^{++}$ p<0.01, $^{+++}$ p<0.001 and $^{++++}$ p<0.0001 for carbachol vs control for each of WT or T1-T5 (two way ANOVA with Tukey post hoc test).

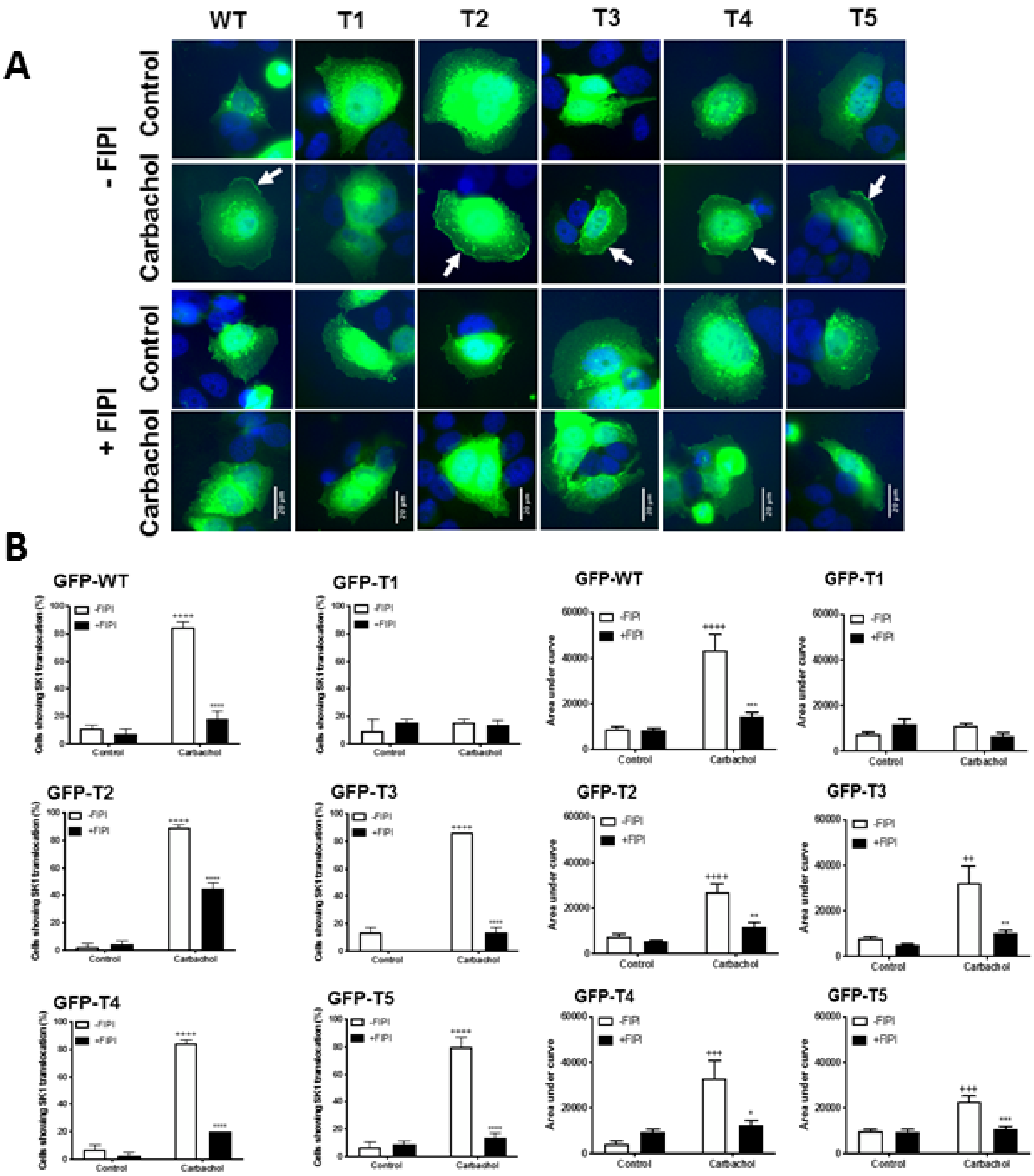

Fig. S4 Sensitivity of T1-T5 mutants to the PLD inhibitor, FIPI in response to carbachol. MCF-7L cells over-expressing WT-GFP-*m*SK1 or T1-T5 mutants were treated with FIPI (100 nM, 60 min) prior to carbachol (100 µM) for 10 min. Cells were processed and mounted with DAPI to stain DNA (blue). **(A)** 40x oil magnification photomicrographs of cells over-expressing WT GFP-*m*SK1 and T1-T5 GFP-*m*SK1 detected with GFP. Representative results of 3 independent experiments. **(B)** The bar graphs represent the AUC of transfected GFP-*m*SK1 (WT or T1-T5) translocation (n=5) and the % cells containing translocated GFP-*m*SK1 (WT or T1-T5) in response to carbachol (n=3); * p<0.05, ** p<0.01, *** p<0.001 and **** p<0.0001 for carbachol vs carbachol with FIPI and for each of WT GFP-*m*SK1 and T1-T5; $^{++}$ p<0.01, $^{+++}$ p<0.001 and $^{++++}$ p<0.0001 for carbachol vs control for each of WT GFP-*m*SK1 or T1-T5 (two way ANOVA with Tukey post hoc test).

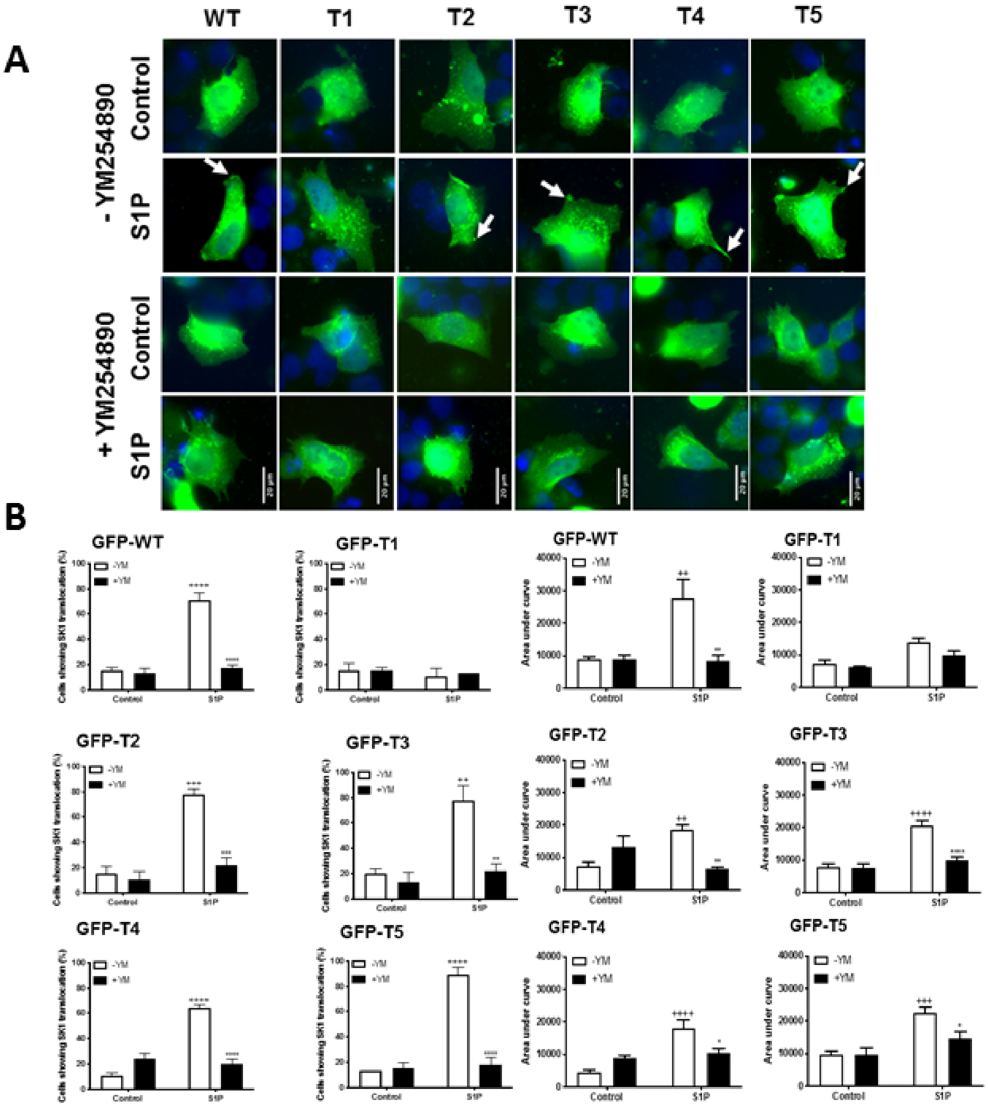

Fig. S5 Sensitivity of T1-T5 mutants to the Gq inhibitor YM254890 in response to S1P. MCF-7L cells over-expressing WT-GFP-mSK1 or T1-T5 mutants were treated with YM254890 (10 µM, 30 min) prior to S1P (5 µM) for 10 min. Cells were processed and mounted with DAPI to stain DNA (blue). **(A)** 40x oil magnification photomicrographs of cells over-expressing WT GFP-*m*SK1 and T1-T5 GFP-*m*SK1 detected with GFP. Representative results of 3 independent experiments. **(B)** The bar graphs represent the AUC of transfected GFP-*m*SK1 (WT or T1-T5) translocation (n=5) and the % cells containing translocated GFP-mSK1 (WT or T1-T5) in response to S1P (n=3); * $p<0.05$, ** $p<0.01$, *** $p<0.001$ and **** $p<0.0001$ for S1P vs S1P with YM254890 and for each of WT GFP-*m*SK1 and T1-T5; $^{++}$ $p<0.01$, $^{+++}$ $p<0.001$ and $^{++++}$ $p<0.0001$ for S1P vs control for each of WT GFP-*m*SK1 or T1-T5 (two way ANOVA with Tukey post hoc test).

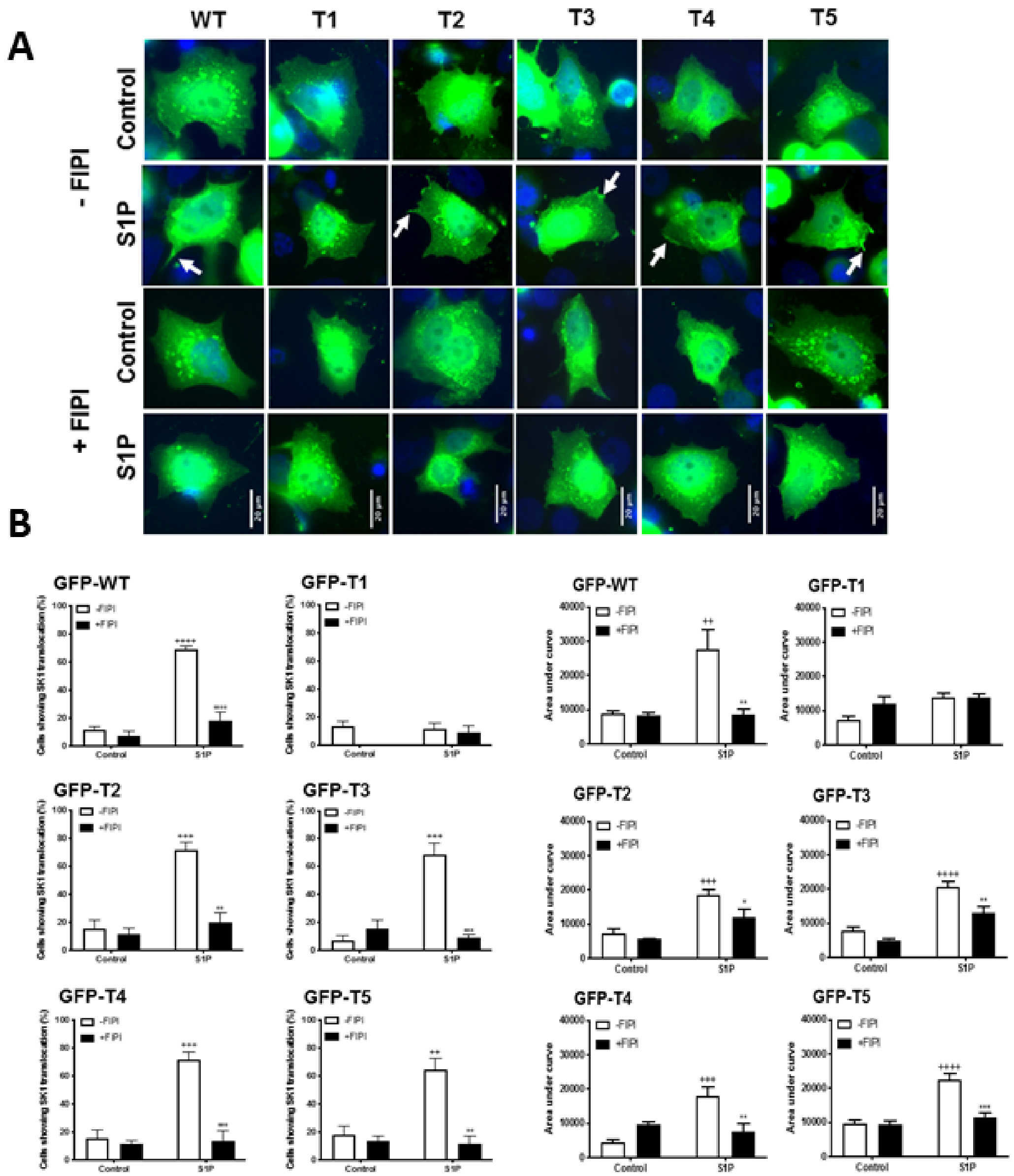

Fig. S6 Sensitivity of T1-T5 mutants to the PLD inhibitor, FIPI in response to S1P. MCF-7L cells over-expressing WT-GFP-*m*SK1 or T1-T5 mutants were treated with FIPI (100 nM, 60 min) prior to S1P (5 µM) for 10 min. Cells were processed and mounted with DAPI to stain DNA (blue). **(A)** 40x oil magnification photomicrographs of cells over-expressing WT GFP-*m*SK1 and T1-T5 GFP-*m*SK1 detected with GFP. Representative results of 3 independent experiments. **(B)** The bar graphs represent the AUC of transfected GFP-*m*SK1 (WT or T1-T5) translocation (n=5) and the % cells containing translocated GFP-*m*SK1 (WT or T1-T5) in response to S1P (n=3); * $p<0.05$, ** $p<0.01$, *** $p<0.001$ and **** $p<0.0001$ for S1P vs S1P with FIPI and for each of WT GFP-*m*SK1 and T1-T5; $^{++}$ $p<0.01$, $^{+++}$ $p<0.001$ and $^{++++}$ $p<0.0001$ for S1P vs control for each of WT GFP-*m*SK1 or T1-T5 (two way ANOVA with Tukey post hoc test).
